# Supplementary material for: A heterozygous CEBPA mutation disrupting the bZIP domain in a RUNX1 and SRSF2 mutational background causes MDS disease progression
Source: Nat Commun. 2025 Jul 1;16:5489. doi: 10.1038/s41467-025-60192-8 (PMC12219322; doi:10.1038/s41467-025-60192-8)
Supplement: Supplementary file 1 — Supplementary Information [file 41467_2025_60192_MOESM1_ESM.pdf]

Supplementary Figure 1

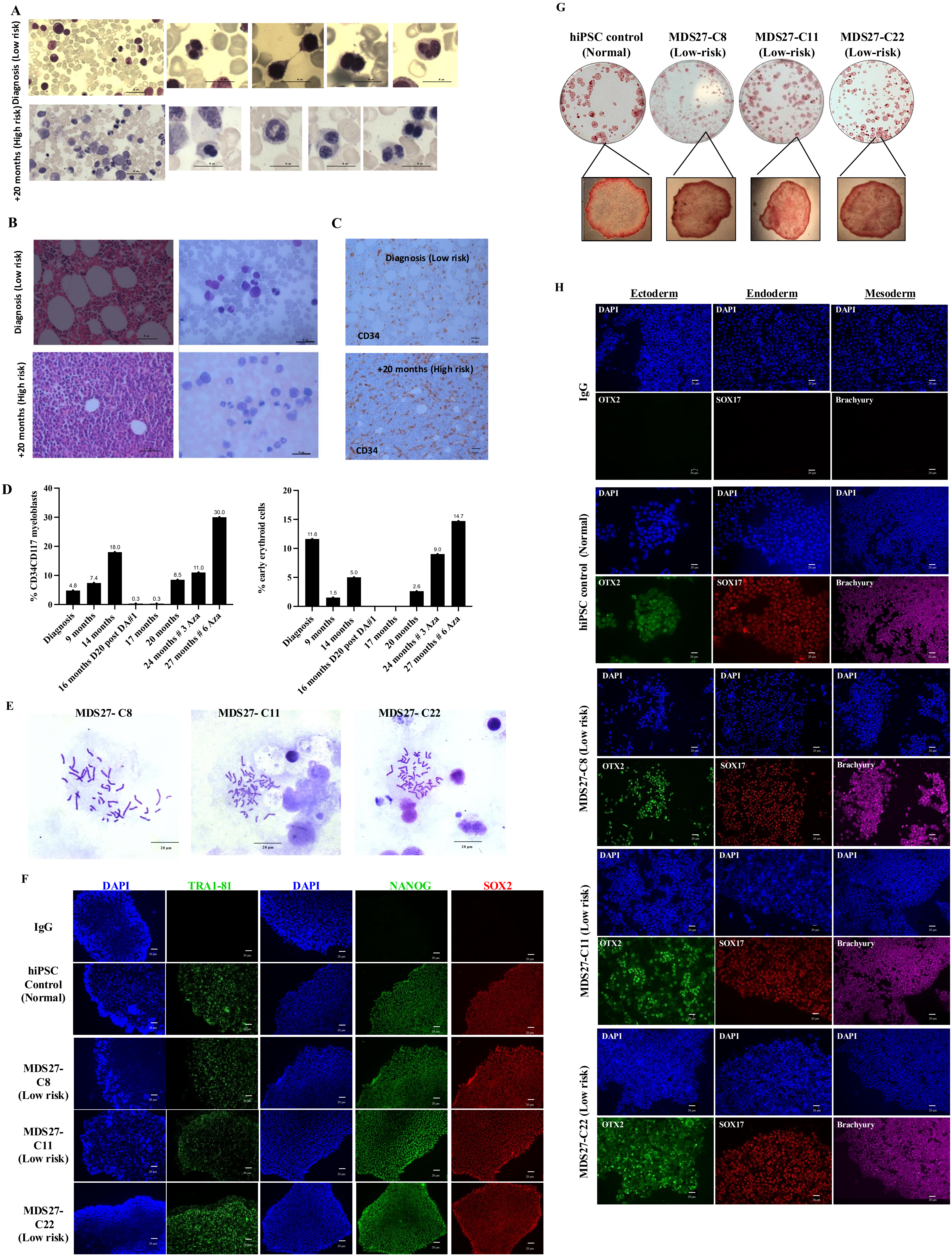

Supplementary Figure 1: MDS27 patient: Clinical characteristics and successful generation of iPSC.

(A) May Grunwald Giemsa staining of bone marrow smears from patient MDS27 at the time of diagnosis (low-risk) and 20 months after diagnosis (high risk). 10 µm scale bar; 63 x magnification. Leica DM6000 light microscope.

(B) Hematoxylin & Eosin (H&E) staining of trephine samples from patient MDS27 at the time of diagnosis (low-risk) and 20 months after diagnosis (high risk). 40x and 63x magnification. Leica DM6000 light microscope.

(C) CD34 immunostaining of trephine samples from patient MDS27 at the time of diagnosis (low-risk) and 20 months after diagnosis (high risk). Leica DM6000 light microscope; 20x magnification.

(D) Bargraphs showing the percentage of blasts (CD34+CD117+) (left) and percentage of early erythroid progenitors (CD71+ CD235a-) of MDS27 patient at different times during disease progression.

(E) Chromosome spreads: Human iPSC clones generated from MDS27 showing a normal number of chromosomes. (Data represent 3 independent experiments: >25 metaphases per sample per experiment), 20 µm scale bar, 100x magnification, Leica DM6000 light microscope.

(F) Representative immunofluorescence image for the staining of hiPSC control and MDS-iPSC colonies with pluripotency markers. DAPI staining is shown in blue. Scale bars, 20 µm. 20x magnification, Leica DM6000 light microscope. N=4 independent experiments.

(G) Positive expression of early pluripotent marker (alkaline phosphatase) in hiPSC control and MDS27-hiPSC lines. 10x magnification, primo vert microscope (ZEISS). N= 3 independent experiments.

(H) Immunofluorescence staining of hiPSC control and MDS27-iPSC with lineage markers. The colour of SOX17 and Brachyury was modified to Red and Purple respectively using ImageJ software. 20 µm scale bar, 20x magnification, Leica DM6000. N= 4 independent experiments.

Supplementary Figure 2

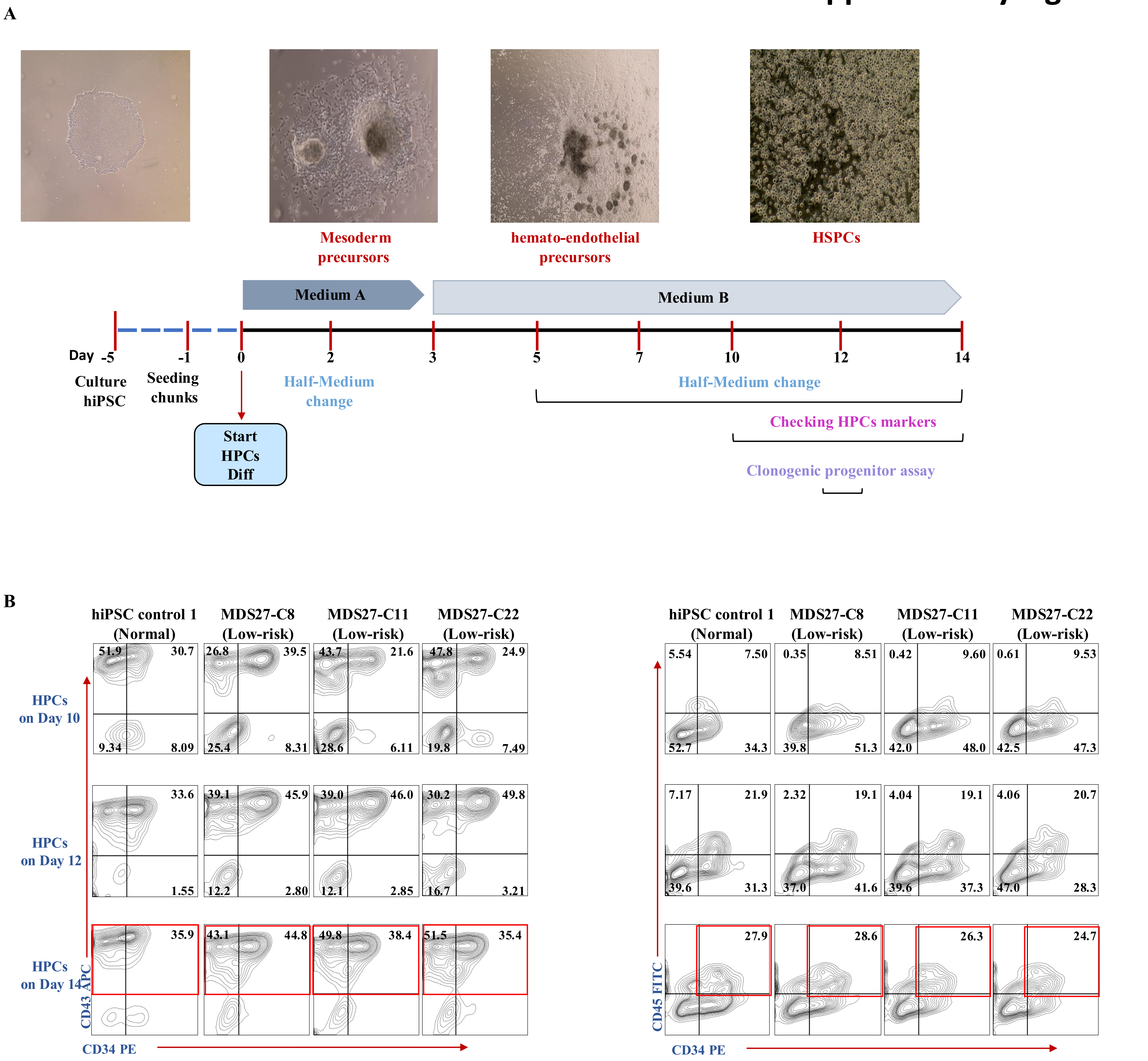

Supplementary Figure 2

C

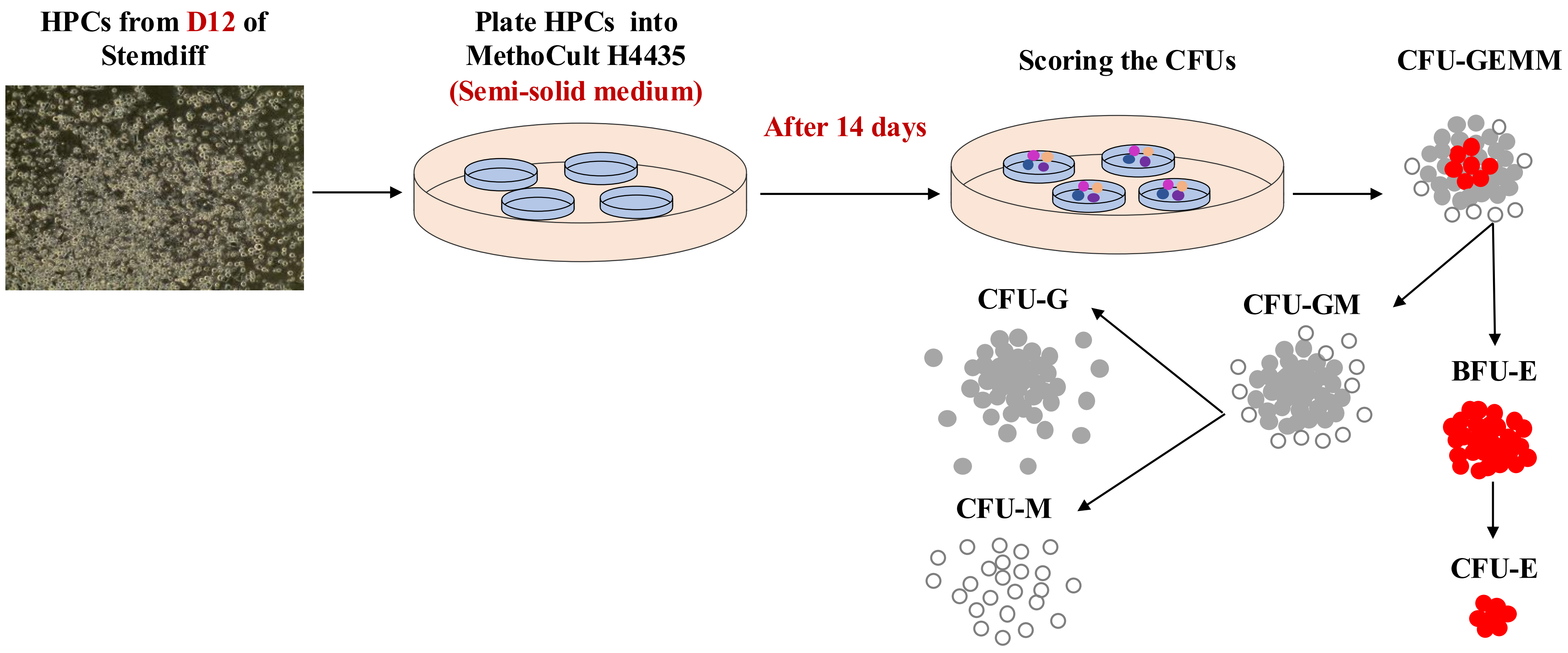

D

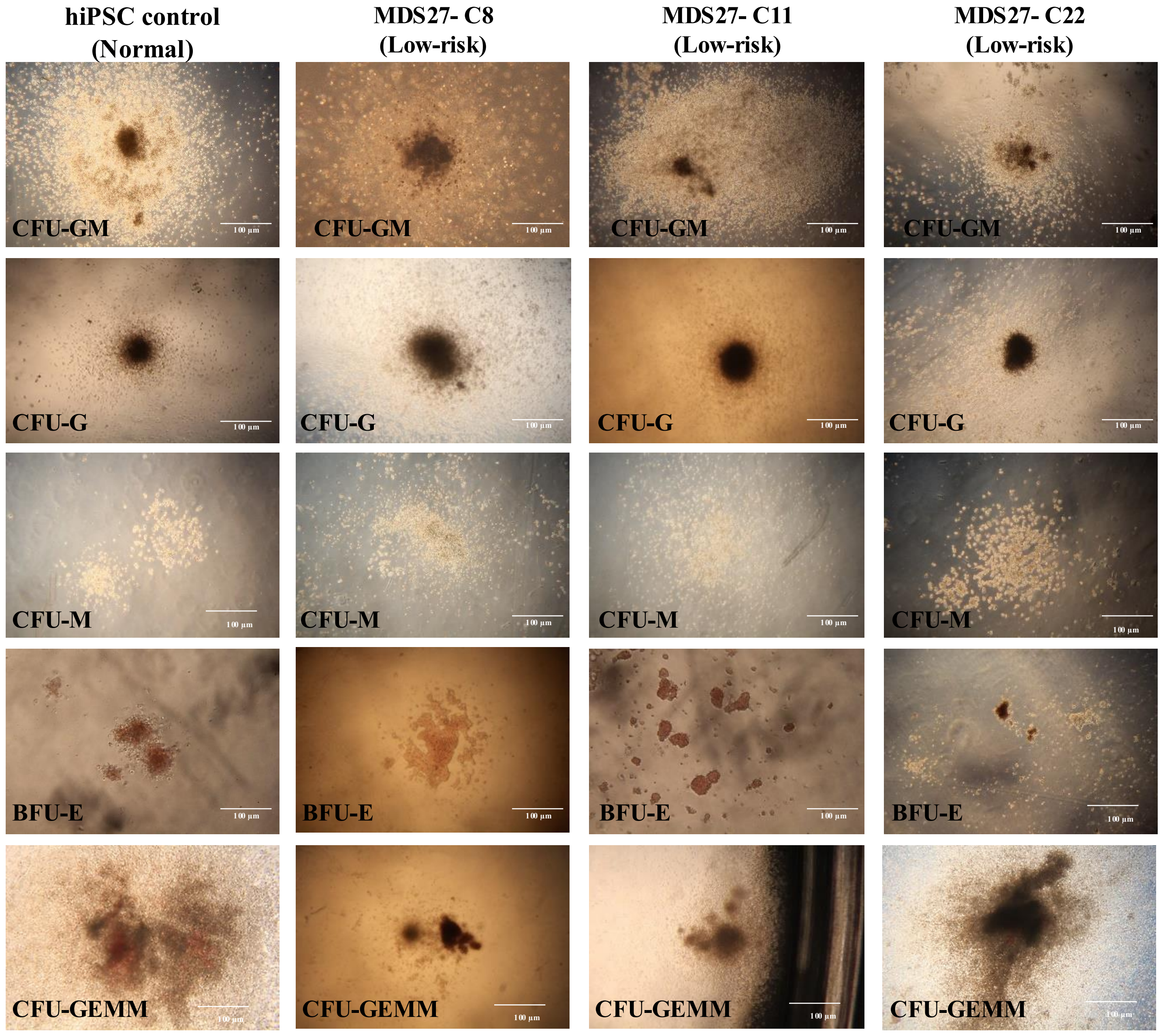

Supplementary Figure 2

E

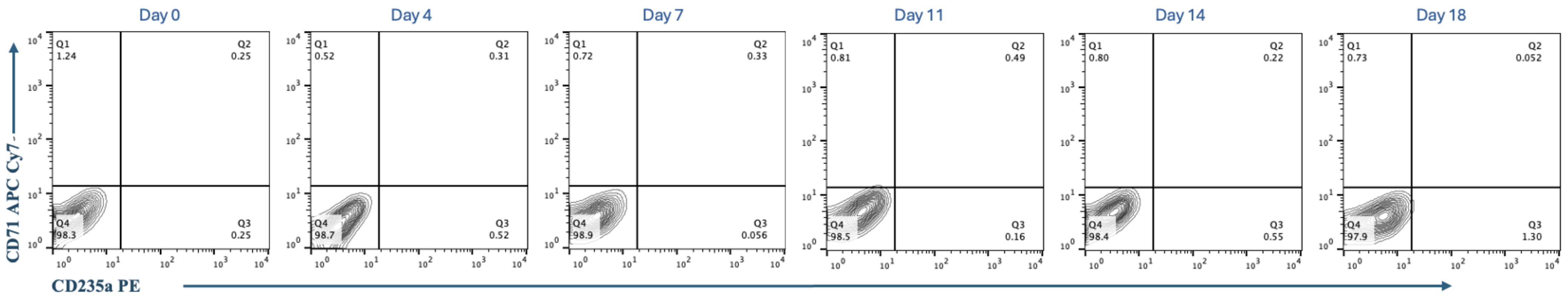

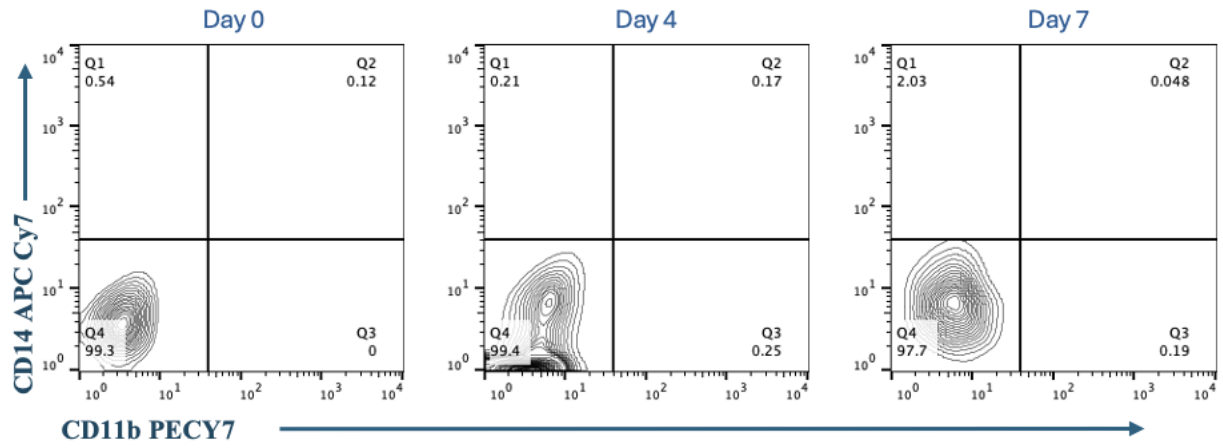

F

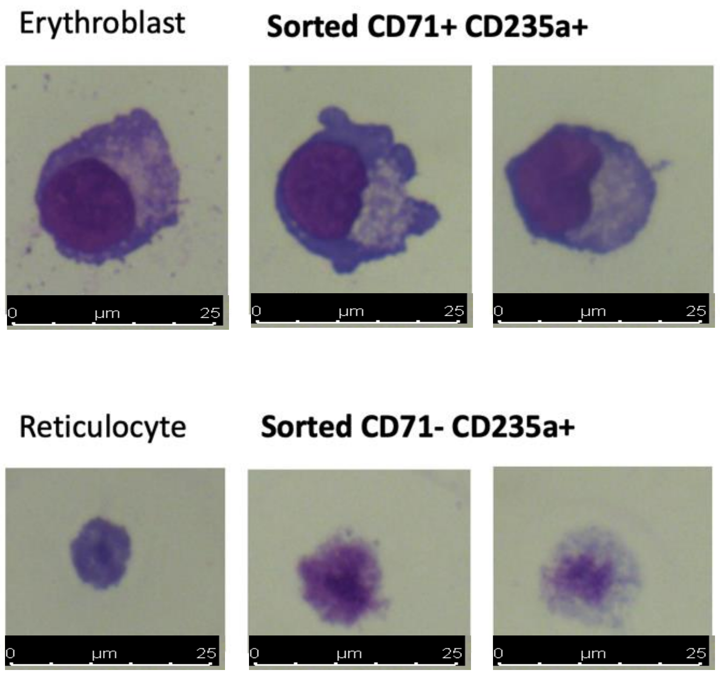

G

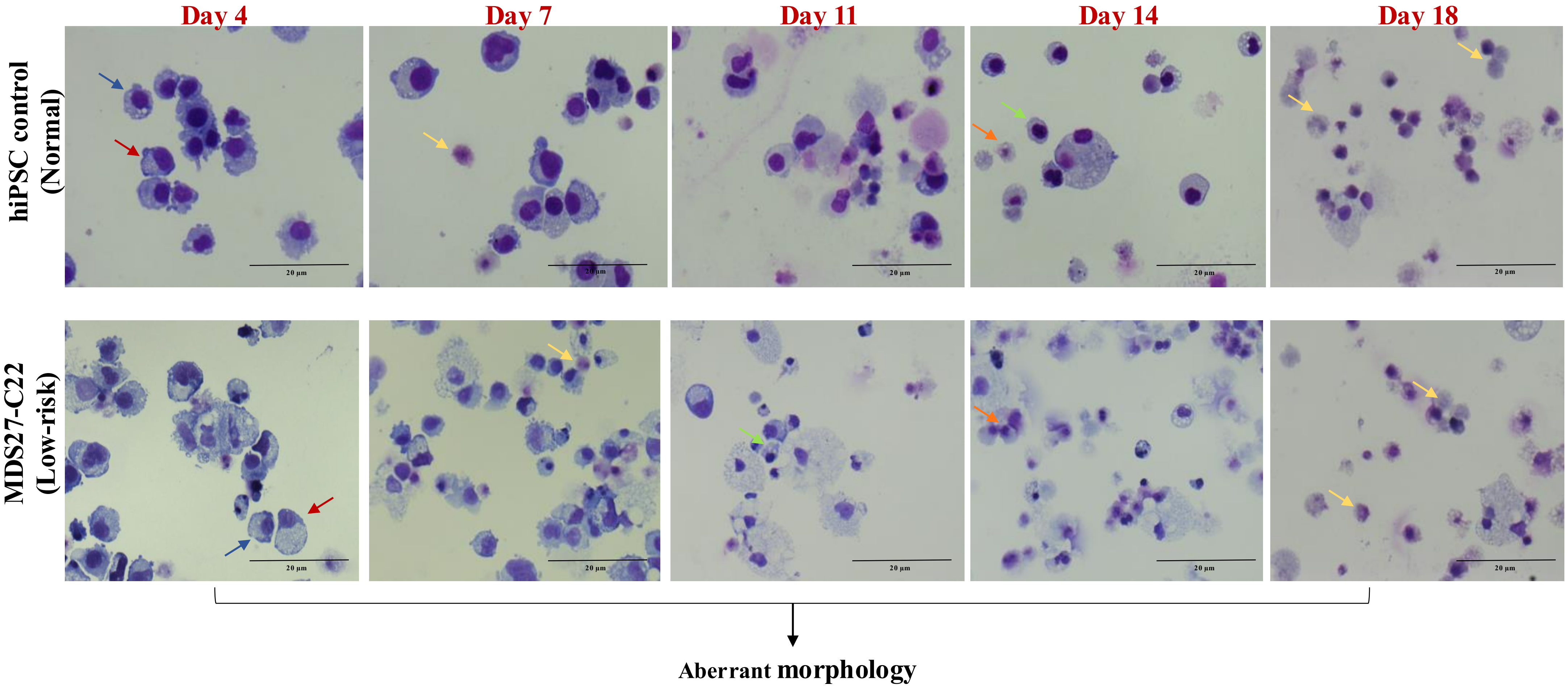

Supplementary Figure 2: *SRSF2 and RUNX1* mutations in MDS27-hiPSCs clones do not affect the HPCs differentiation

(A) Schematic representation of the HPC differentiation using hematopoietic STEMdiff protocol from stem cell technology.

(B) Representative images of flow cytometric analysis of CD43+ and $CD34^{+}$ $CD45^{+}$ at different times during heamatopoietic differentiation (Day 10, 12 and 14). N=4 independent experiments.

(C) Schematic representation of the experimental procedure for the colony forming assays. Artwork generated with powerpoint Bundle-Biology.

(D) Representative images of the morphology of the different CFU scored at 14 days in semi-solid medium. Pictures taken with primo vert microscope (Zeiss) with Canon camera at 4x magnification. Scale bar 100 µm. N=4 independent experiments.

(E) Contour plots for isotype controls used on erythroid and myeloid analysis.

(F) Diffquick stained cytospins of sorted erythroid CD71+/CD235a+ cells and CD71-/CD235a+ cells. The pictures were taken Leica DM6000 at 100x magnification, 25 µm scale bar.

(G) Representative images of Diffquick stained cytospins of erythroid cells grown in liquid culture at the times indicated. Morphological analysis of erythroid cells shows the maturation stages during different days of the culture: Proerythroblast (red arrow), Basophilic erythroblast (blue arrow), Polychromatic erythroblast (green arrow), Orthochromatic erythroblast (orange arrow) & Mature cells (yellow arrow). The pictures were taken with a Leica DM6000 at 40x magnification, 20 µm scale bar. N= 4 independent experiments.

Supplementary Figure 3

A

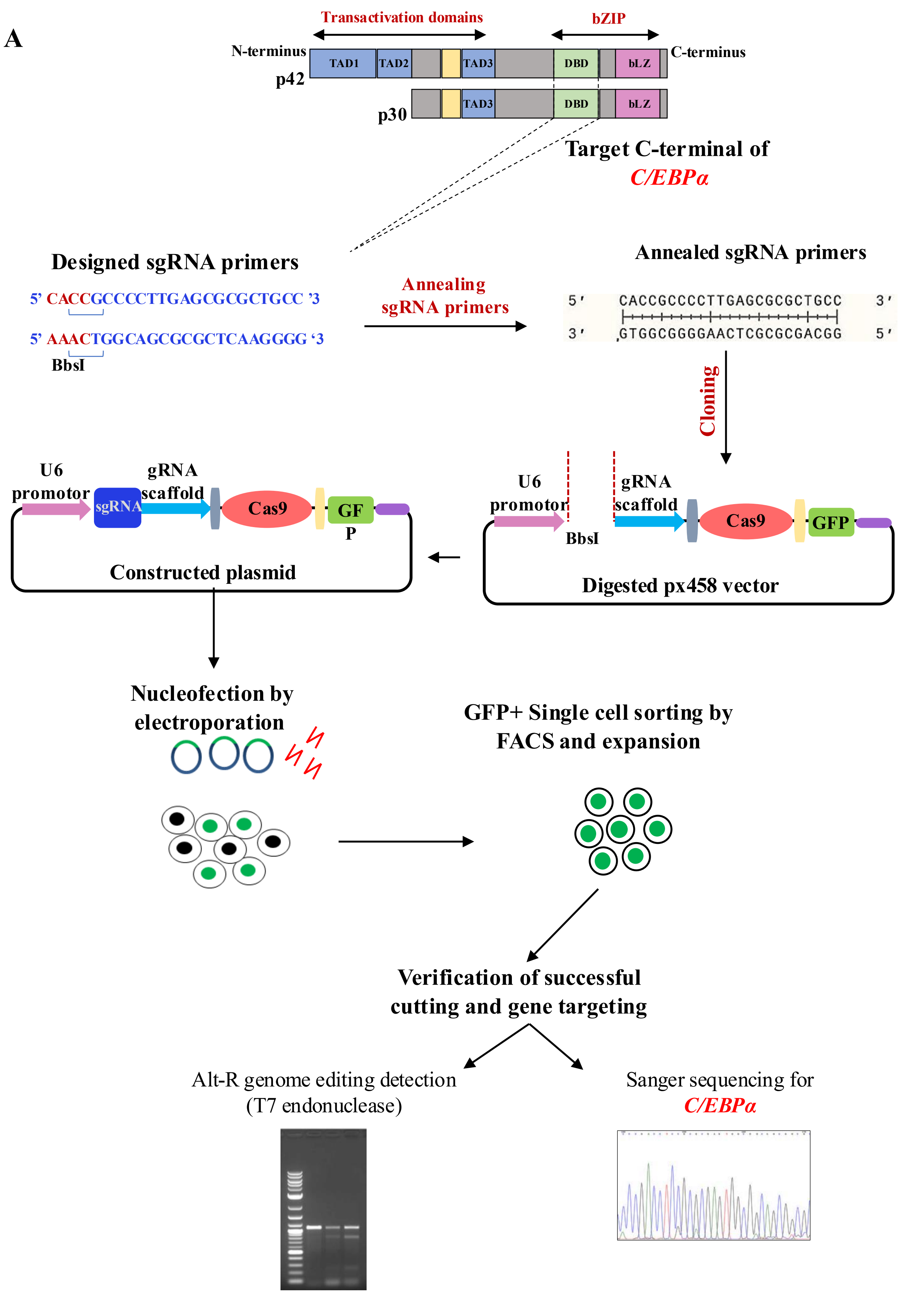

B

WT *C/EBPα* sequence

CGGGGGAGCGCACGGGCCCCCGCCCGGCTACGGCTGCGCGGCCGCCGGCTACCTGGA
CGGCAGGCTGGAGCCCCTGTACGAGCGCGTCGGGGCGCCGGCGCTGCGGCCGCTGGT
GATCAAGCAGGAGCCCCGCGAGGAGGATGAAGCCAAGCAGCTGGCGCTGGCCGGCCT
CTTCCCTTACCAGCCGCCGCCGCCGCCGCCGCCCTCGCACCCGCACCCGCACCCGCCG
CCCGCGCACCTGGCCGCCCCGCACCTGCAGTTCCAGATCGCGCACTGCGGCCAGACCA
CCATGCACCTGCAGCCCGGTCACCCCACGCCGCCGCCCACGCCCGTGCCCAGCCCGCA
CCCCGCGCCCGCGCTCGGTGCCGCCGGCCTGCCGGGCCCTGGCAGCGCGCTCAAGGGG
CTGGGCGCCGCGCACCCCGACCTCCGCGCGAGTGGCGGCAGCGGCGCGGGCAAGGCC
AAGAAGTCGGTGGACAAGAACAGCAACGAGTACCGGGTGCGGCGCGAGCGCAACAA
CATCGCGGTGCGCAAGAGCCGCGACAAGGCCAAGCAGCGCAACGTGGAGACGCAGCA
GAAGGTGCTGGAGCTGACCAGTGACAATGACCGCCTGCGCAAGCGGGTGGAACAGCT
GAGCCGCGAACTGGACACGCTGCGGGGCATCTTCCGCCAGCTGCCAGAGAGCTCCTTG
GTCAAGGCCATGGGCAACTGCG

Yellow → **Mutant part in MDS27**

Red → **Primers used to amplify the target region**

Pink → **Position of gRNA**

C

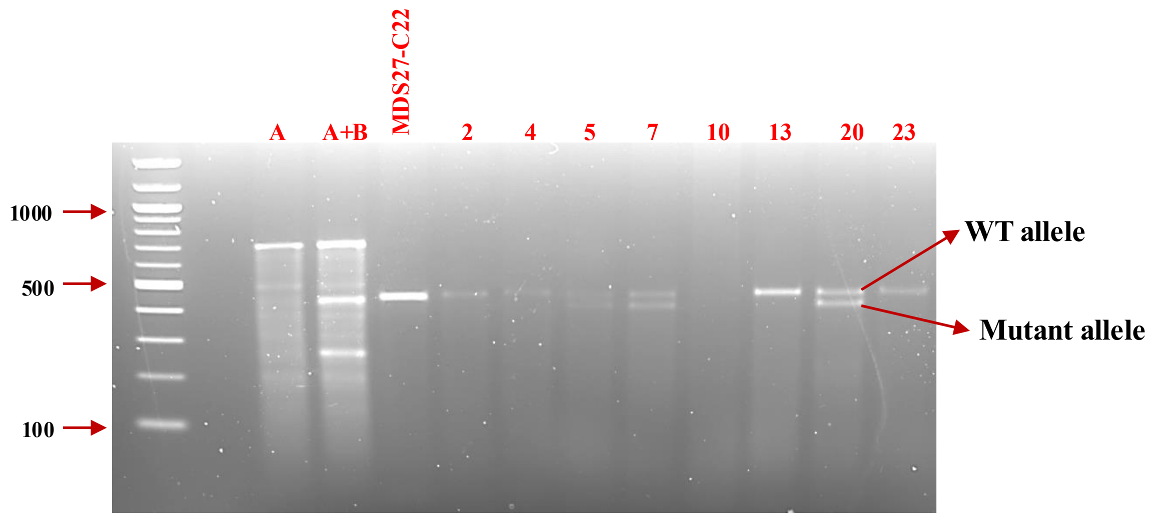

D

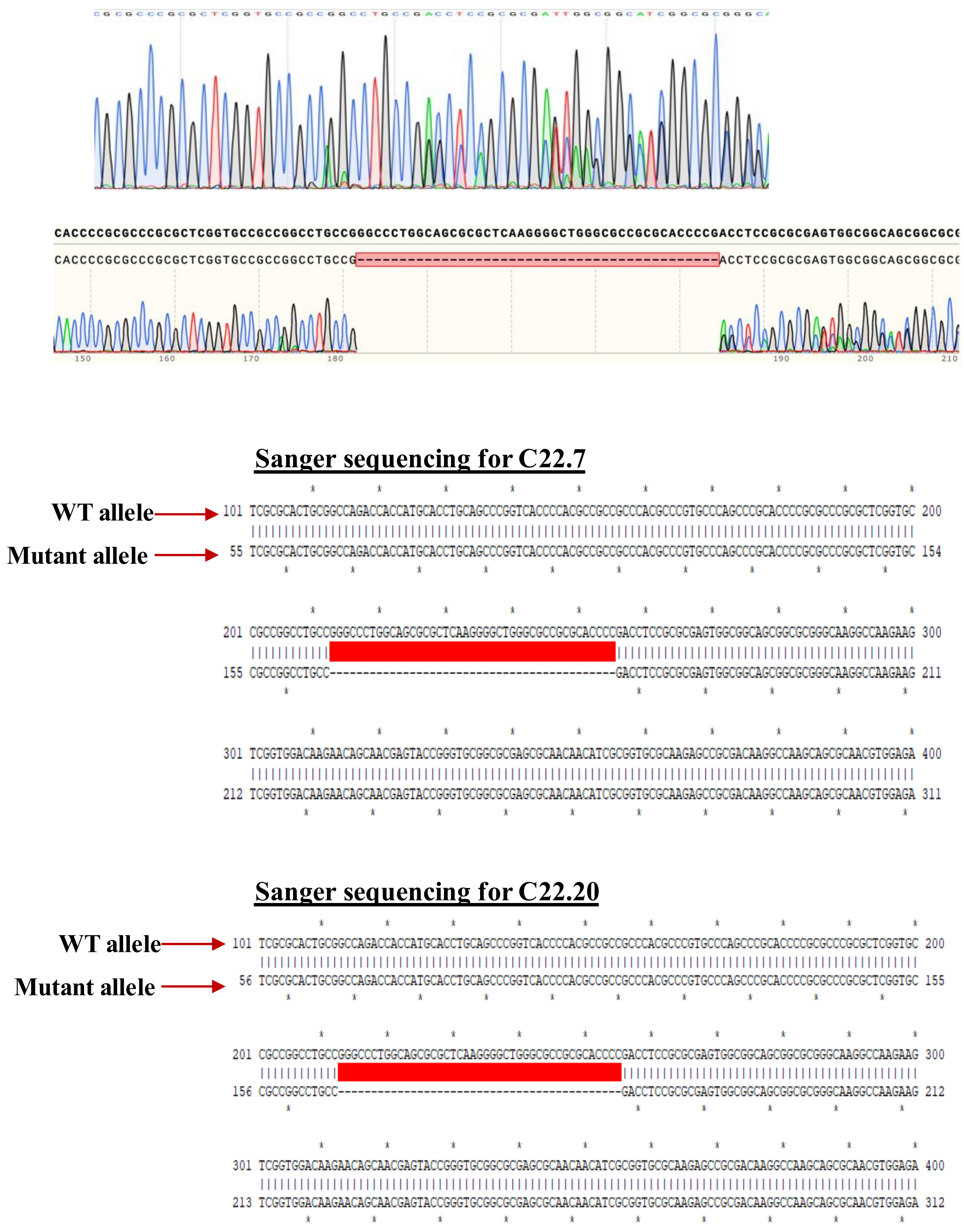

E

WT full *C/EBPα* protein sequence

M E S A D F Y E A E P R P P M S S H L Q S P P H A P S S A A F G F P R G A
G P A Q P P A P P A A P E P G G I C E H E T S I D I S A Y I D P A A F N D E F
L A D L F Q H S R Q Q E K A K A A V G P T G G G G G GD F D Y P G A P A
G P G G A M P G G A H G P P P G Y G C A A A G Y L D G R L E P L Y E R V
G A P A LR P L V I K Q E P R E E D E A K Q L A L A G L F P Y Q P P P P P P
P S H P H P H P P P A H L A A P H L Q FQ I A H C G Q T T M H L Q P G H P
T P P P T P V P S P H P A P A L G A A G L P G P G S A L K G L G A AH P D L
R A S G G S G A G K A K K S V D K N S N E Y R **V R R E R N N I A V R K S**
R D K A K Q R N VE T Q Q K V L E **L T S D N D R L R K R V E Q L S R E**
L D T L R G I F R Q L P E S S L V K A M G N C A

MDS27-Patient *C/EBPα* protein sequence

P H P P P A H L A A P H L Q F Q I A H C G Q T T M H L Q P G H P T P P P T
P V P S P H P A P A L G A A G L P G P S A L K G L A G R R A P R P P R E W
R Q R R G Q G Q E VG G Q E Q Q R V **P G A A R A Q Q H R G A Q E P R Q**
G Q A A Q R G D A A E G A **G A D Q** *

MDS27-C22.7 and C22.20 *C/EBPα* protein sequence

P H P P P A H L A A P H L Q F Q I A H C G Q T T M H L Q P G H P T P P P T
P V P S P H P A P A L G A A G L P T S A R V A A A A R A R P R S R W T R T
A T S T G **C G A S A T T S R C A R A A T R P S S A** T W R R S R R C W S *

Pink → **WT aa**

Grey → **MUTANT aa**

SEQUENCE FOR DNA BINDING DOMAIN

SEQUENCE FOR DIMERIZATION DOMAIN

Supplementary Figure 3

F

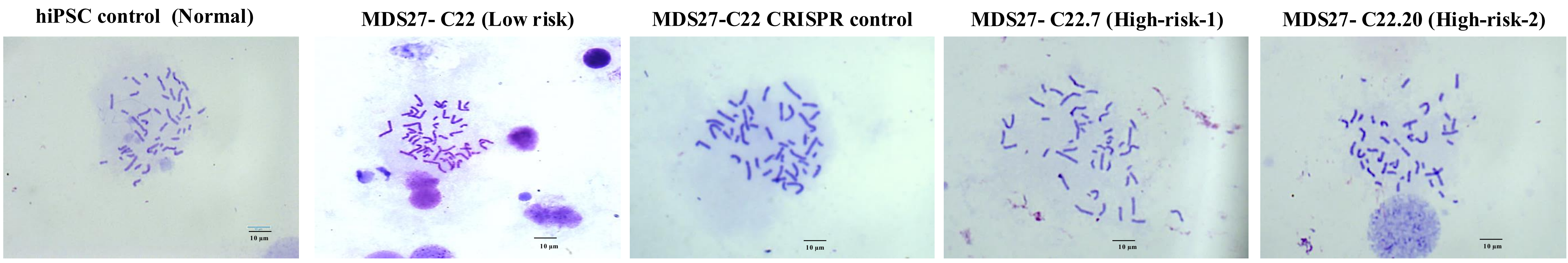

G

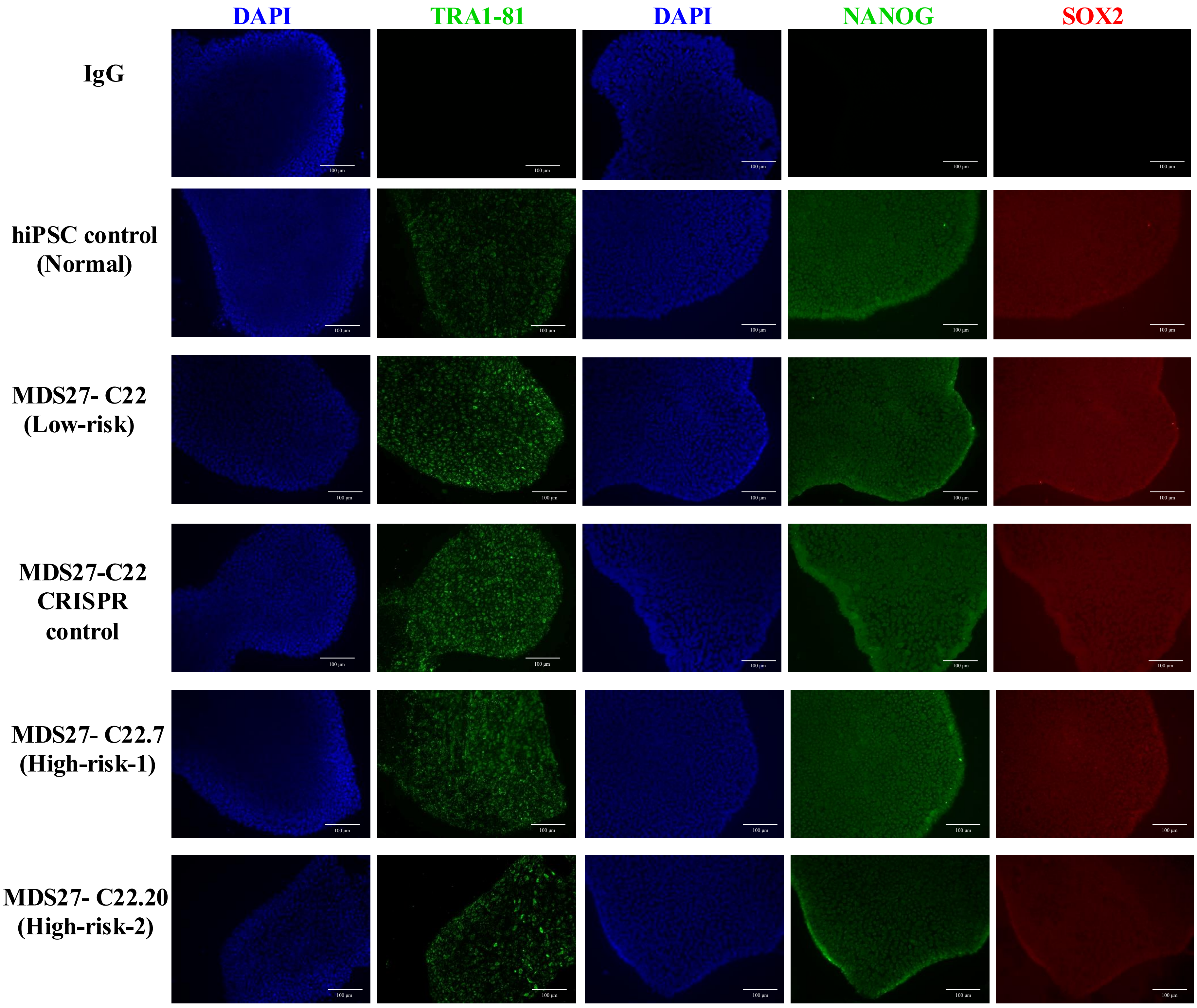

Supplementary Figure 3: Generation and characterization of high-risk MDS27 isogenic clones by CRISPR-Cas9

(A) Schematic representation of engineering *C/EBPα* mid region mutation in MDS27-C22 using PX458 Cas9 plasmid. Guide RNA targeted mid region of C/EBPα were designed via an online tool (Trust Sanger Institute Editing database). Then, the sgRNA guide was cloned into an expression plasmid bearing both sgRNA scaffold backbone (BB) and Cas9, pSpCas9(BB) (PX458). The constructed plasmid was transfected into hiPSC MDS27-C22. T7I endonuclease assay and Sanger sequencing confirmed the generation of *C/EBPα* mutation. Artwork generated with powerpoint Bundle-Biology.

(B) Snapshot of C/EBPα sequence centre in the mutated region. Position of gRNA (pink), primers used to amplify the targeted region (red) and the deleted nucleotides (blue) after CRISPR-Cas9 are highlighted.

(C) Mismatch cleavage assay T7EI was performed in different isolated clones. The Digestion reactions were analysed on an agarose gel. Sample 1 contains Control A homoduplexes PCR products, while Sample 2 contains homoduplexes and heteroduplexes of Control A and B PCR products. Expected DNA fragment size is indicated as WT, and that showing a deletion indicated as mutant allele.

(D) Dendrograms and sequence alignment after Sanger sequencing of the clones (MDS27-C22.7 and MDS27-C22.20) with CEBPA mutation.

(E) CEBPA protein sequence from WT, MDS27 patient and iPSC high risk clones (MDS27-C22.7 and MDS27-C22.20).

(F) Chromosome spreads showing normal number of chromosomes for the indicated iPSCs lines. (>25 metaphases per sample per experiment), 10µm scale bar, 100x magnification, Leica DM6000 light microscope.

(G) Representative immunofluorescence images after staining with pluripotency markers. DAPI staining is shown in blue. Scale bars, 100 µm; 20x magnification, Leica DM6000 light microscope (n=3 independent experiments).

Supplementary Figure 4

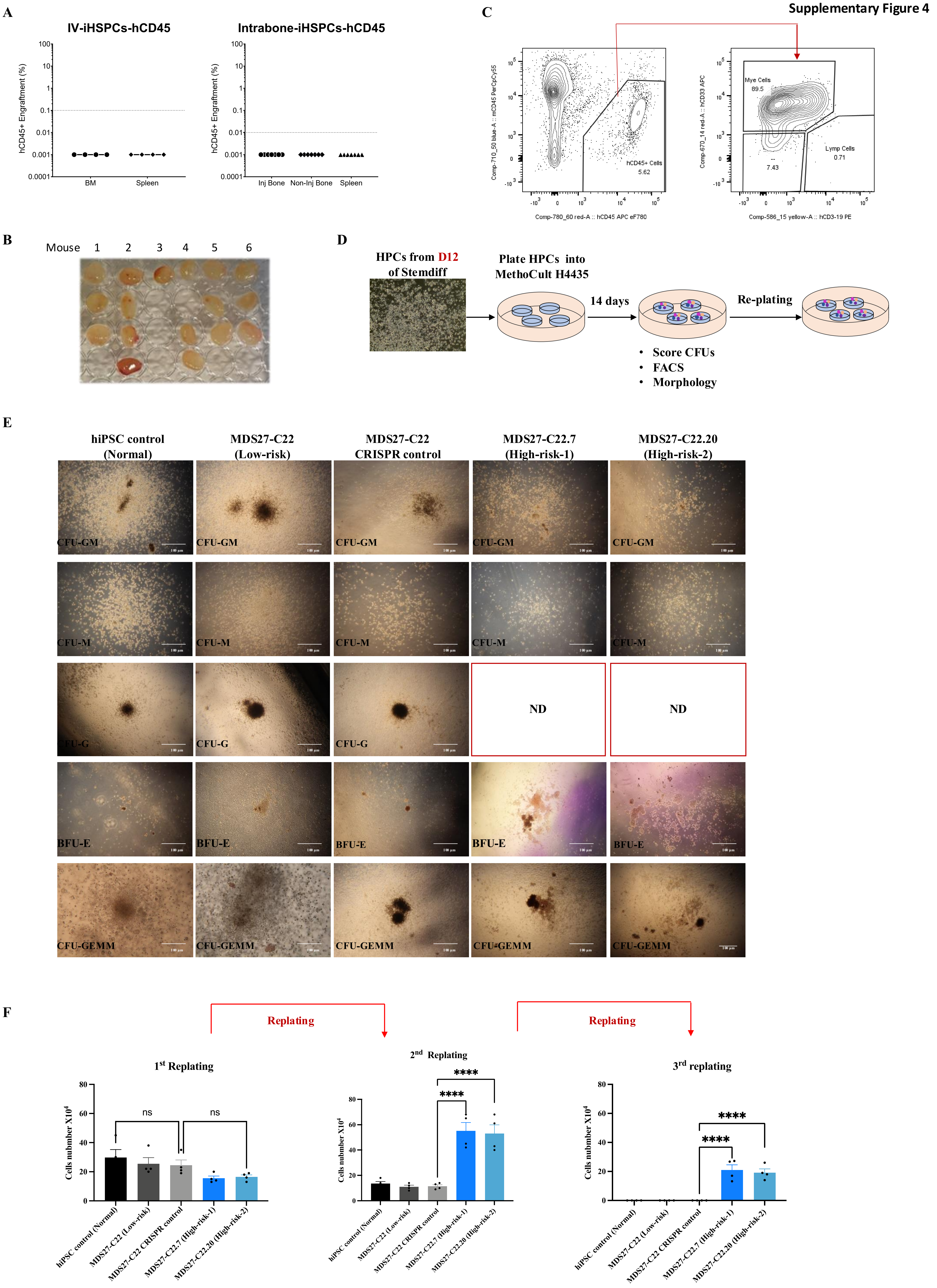

Supplementary Figure 4: Further potential differentiation of HPCs in semi-solid medium

(A) Percentage of $hCD45^+$ cells in murine bone marrow and spleen in NSGW41 mice at week12 (left). iHSPCs from healthy donor and MDS were injected into the mice via intravenous route (n=4). Percentage of hCD45-engrafted cells in the injected bone, non-injected bone and spleen (right). iHSPCs from healthy donor and MDS were injected into the mice via intrabone route (n=7). Please see Source of data file.

(B) Humanised scaffolds retrieved from mice. Each mouse was transplanted with 1 to 4 scaffolds.

(C) Representative flow cytometry plot of the cells retrieved from the humanised niches following the xenotransplantation. Mye- Myeloid cells, Lymp- Lymphoid cells.

(D) Schematic representation of the experimental design. Artwork generated with powerpoint Bundle-Biology.

(E) Representative images illustrating the morphology of CFUs scored after 14 days in semi-solid medium. Pictures taken with primo vert microscope (Zeiss) with Canon camera at 4x magnification; scale bar 100 μm. n=4 independent experiments.

(F) Number of the single cells that obtained after each replating. Statistical results are presented as mean ± SEM. ns= no significant *p <0.05, ****p <0.0001, One-way ANOVA with Dunnett's multiple comparisons. N=4 independent experiments. Please see Source of data file and Supplementary Data Table 5 for exact p values.

Supplementary Figure 5

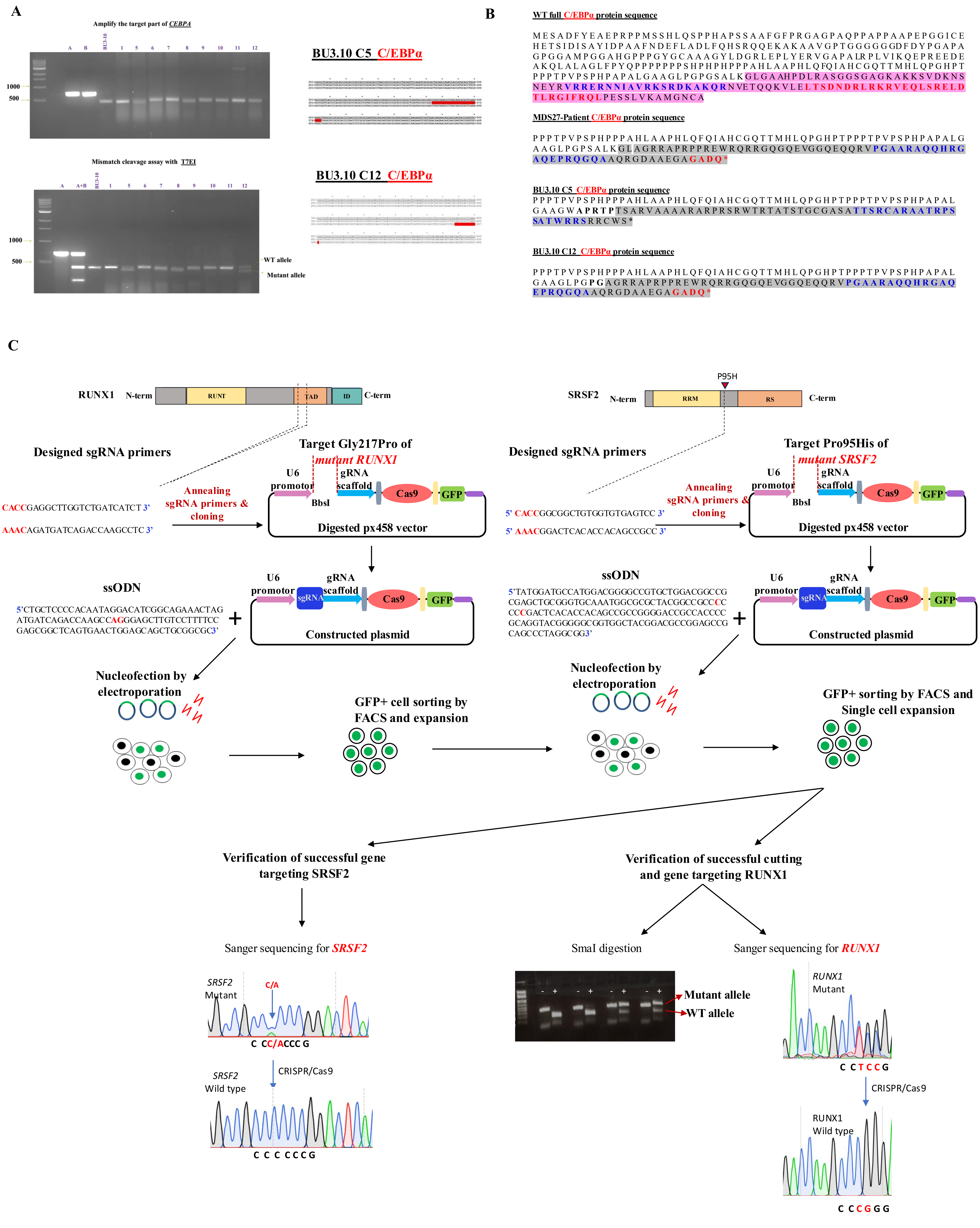

Supplementary Figure 5: CRISPR/Cas9 genome editing to generate a *C/EBPα* bZIP mutation in healthy iPSc and to revert *SRSF2* (P95H) and *RUNX1 (*Gly217Pro-fs) mutations in the MDS27-C22.7 High risk iPSC.

(A) T7I endonuclease assay and Sanger sequencing confirmed the generation of *C/EBPα* mutation. Sequence alignment of wild type allele and mutant alleles from BU3.10- *C/EBPα*^{mut} C5 and C12.

(B) CEBPA protein sequence from WT, MDS27 patient and iPSC BU3.10- *C/EBPα*^{mut} C5 and C12.

(C) Schematic representation of engineering *SRSF2* P95H *and RUNX1* Gly217Pro-fs mutations in MDS27-C22.7 using PX458 Cas9 plasmid. Guide RNAs targeting the specific mutated region as well as as donor DNA primers for each gene were designed via an online tool (Trust Sanger Institute Editing database). Then, the sgRNA guide were cloned into pSpCas9(BB) (PX458). The RUNX1 constructed plasmid together with the ssODN were transfected into hiPSC MDS27-C22.7, 24hours after sorted based on GFP expression, cultured and 72h after transfected with the SRSF2 construct plasmid together with the ssODN. Correction of RUNX1 mutation was assessed by SmaI digestion and Sanger sequencing confirmed the correction of both mutations as shown on the dendograms.

A

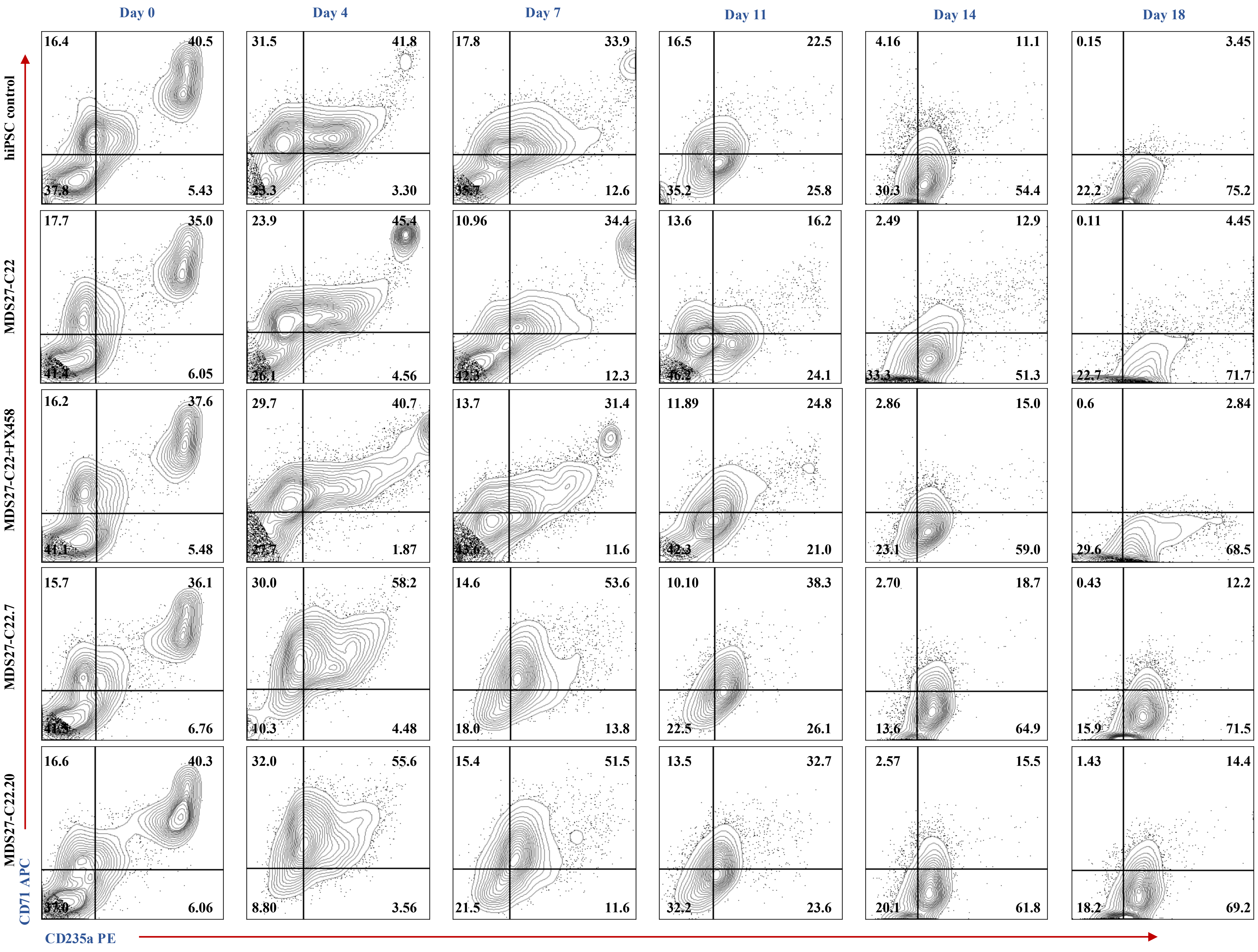

Day 0
Day 4
Day 7
Day 11
Day 14
Day 18
hiPSC control
MDS27-C22
MDS27-C22+PX458
MDS27-C22.7
MDS27-C22.20
CD71 APC
CD235a PE
16.4
40.5
37.8
5.43
31.5
41.8
23.3
3.30
17.8
33.9
35.7
12.6
16.5
22.5
35.2
25.8
4.16
11.1
30.3
54.4
0.15
3.45
22.2
75.2
17.7
35.0
41.4
6.05
23.9
45.4
26.1
4.56
10.96
34.4
42.3
12.3
13.6
16.2
46.2
24.1
2.49
12.9
33.3
51.3
0.11
4.45
22.7
71.7
16.2
37.6
41.1
5.48
29.7
40.7
27.7
1.87
13.7
31.4
43.6
11.6
11.89
24.8
42.3
21.0
2.86
15.0
23.1
59.0
0.6
2.84
29.6
68.5
15.7
36.1
41.5
6.76
30.0
58.2
10.3
4.48
14.6
53.6
18.0
13.8
10.10
38.3
22.5
26.1
2.70
18.7
13.6
64.9
0.43
12.2
15.9
71.5
16.6
40.3
37.0
6.06
32.0
55.6
8.80
3.56
15.4
51.5
21.5
11.6
13.5
32.7
32.2
23.6
2.57
15.5
20.1
61.8
1.43
14.4
18.2
69.2

Supplementary Figure 6

B

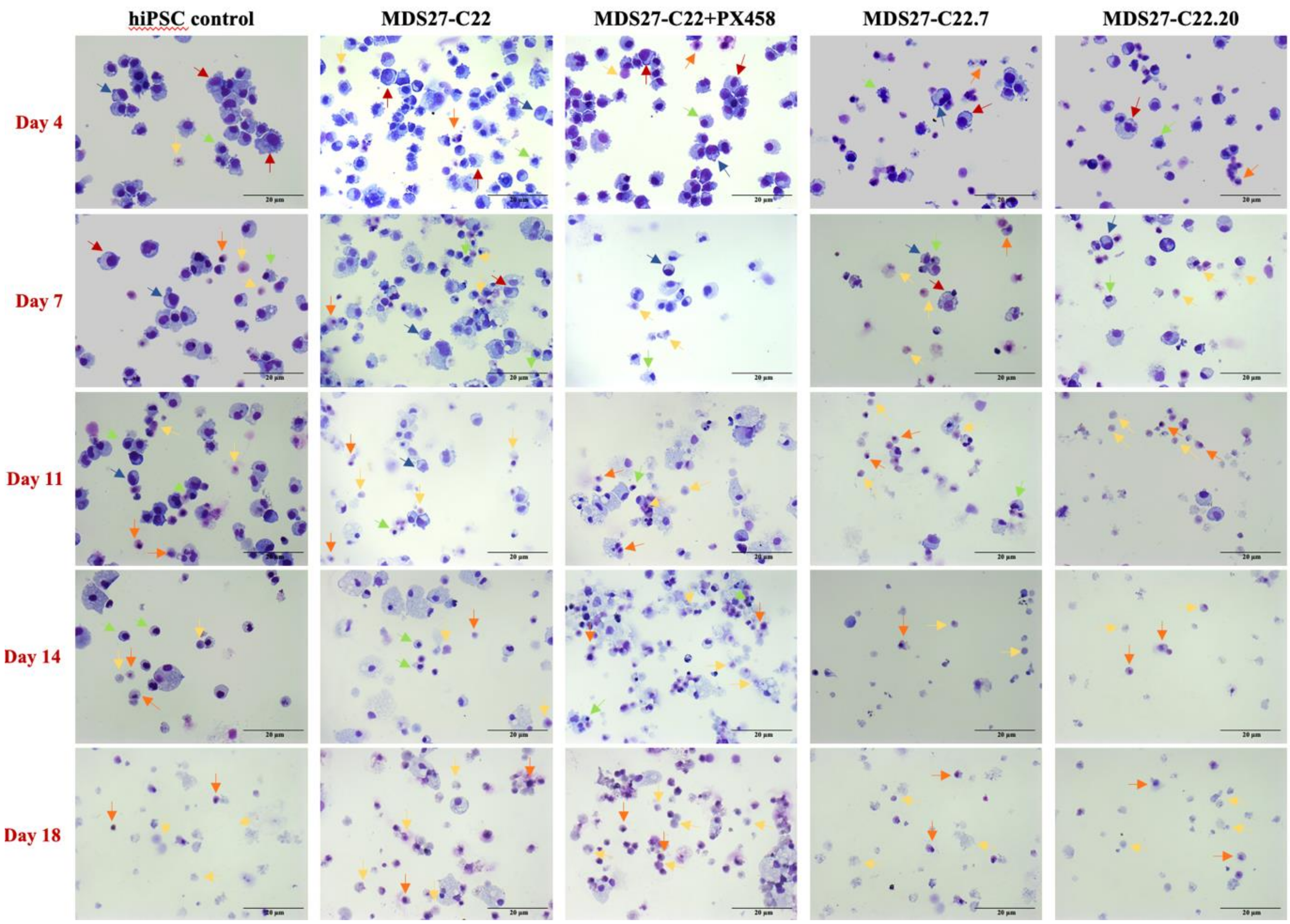

C

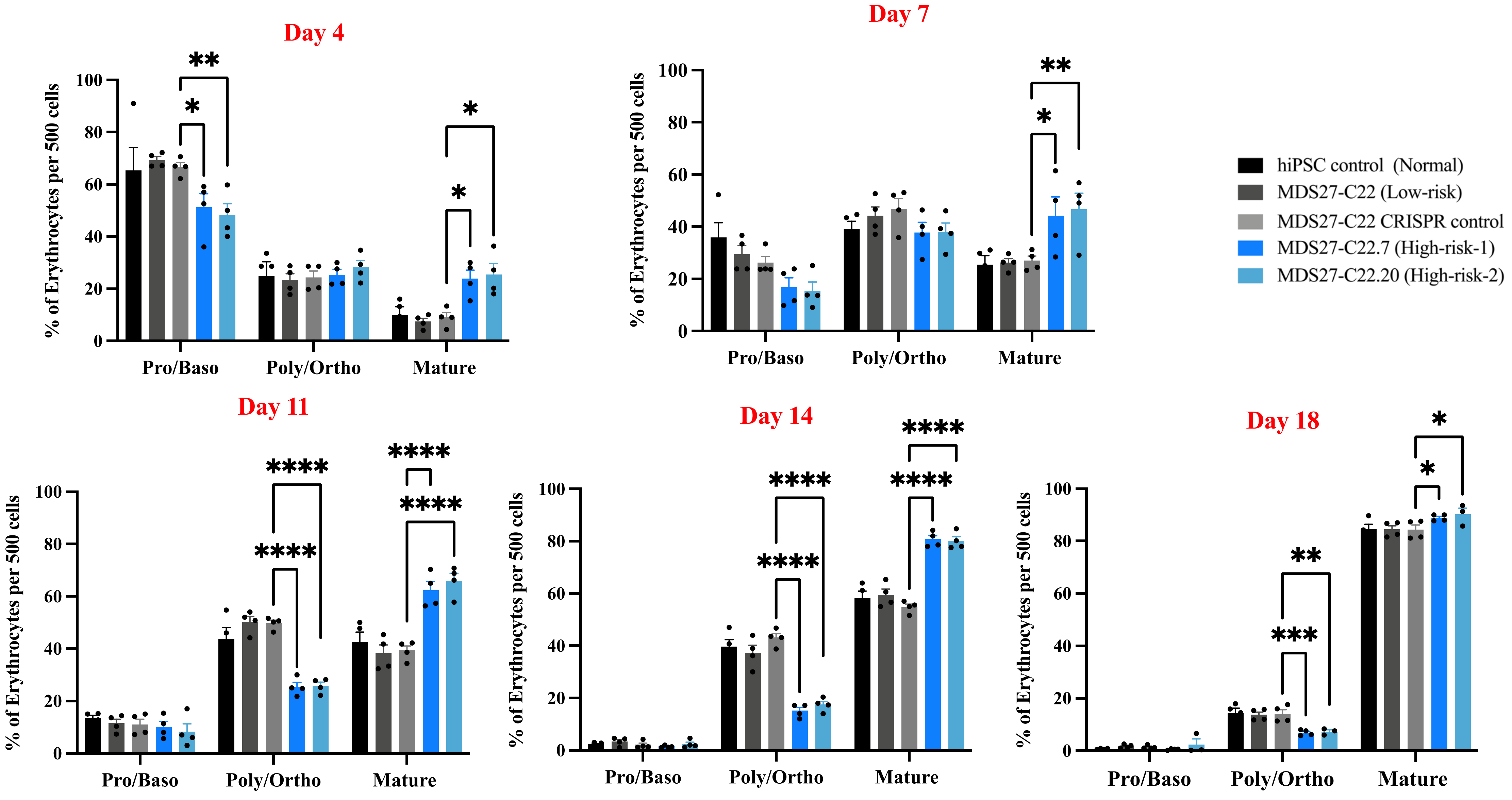

Supplementary Figure 6. Erythroid differentiation timecourse.

(A) Representative dot plots of erythroid differentiation based on CD71 and CD235a expression at different days during erythroid differentiation in liquid culture (Day 4, 7, 11, 14 and 18).

(B) Representative images of Diffquick stained cytospins of erythroid cells grown in liquid culture at the times indicated. Morphological analysis of erythroid cells shows the maturation stages during different days of the culture: Proerythroblast (red arrow), Basophilic erythroblast (blue arrow), Polychromatic erythroblast (green arrow), Orthochromatic erythroblast (orange arrow) & Mature cells (yellow arrow). The pictures were taken Leica DM6000 at 40x magnification, 20 µm scale bar. N= 4 independent experiments.

(C) Bargraphs showing the percentage of erythrocytes at different time points during erythroid per differentiation. 500 cells were counted. Mean and SEM are shown. **** <0.0001, **p<0.001, * p<0.05 and (ns, no significant), Two-way ANOVA with Dunnett's correction. N= 4 independent experiments. Please see Source of data file and Supplementary Data Table 5 for exact p values.

Supplementary Figure 7

A

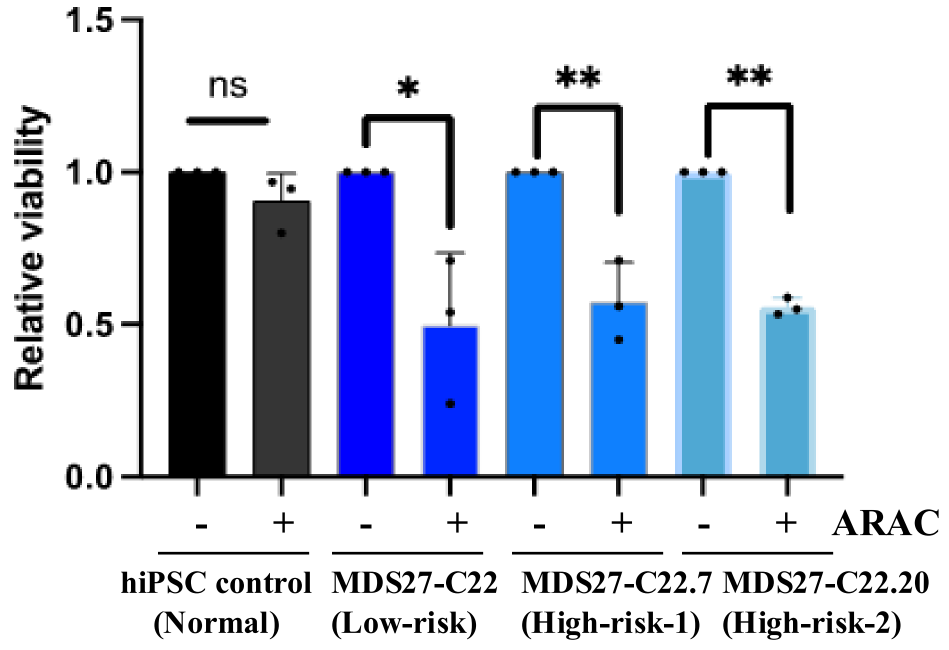

B

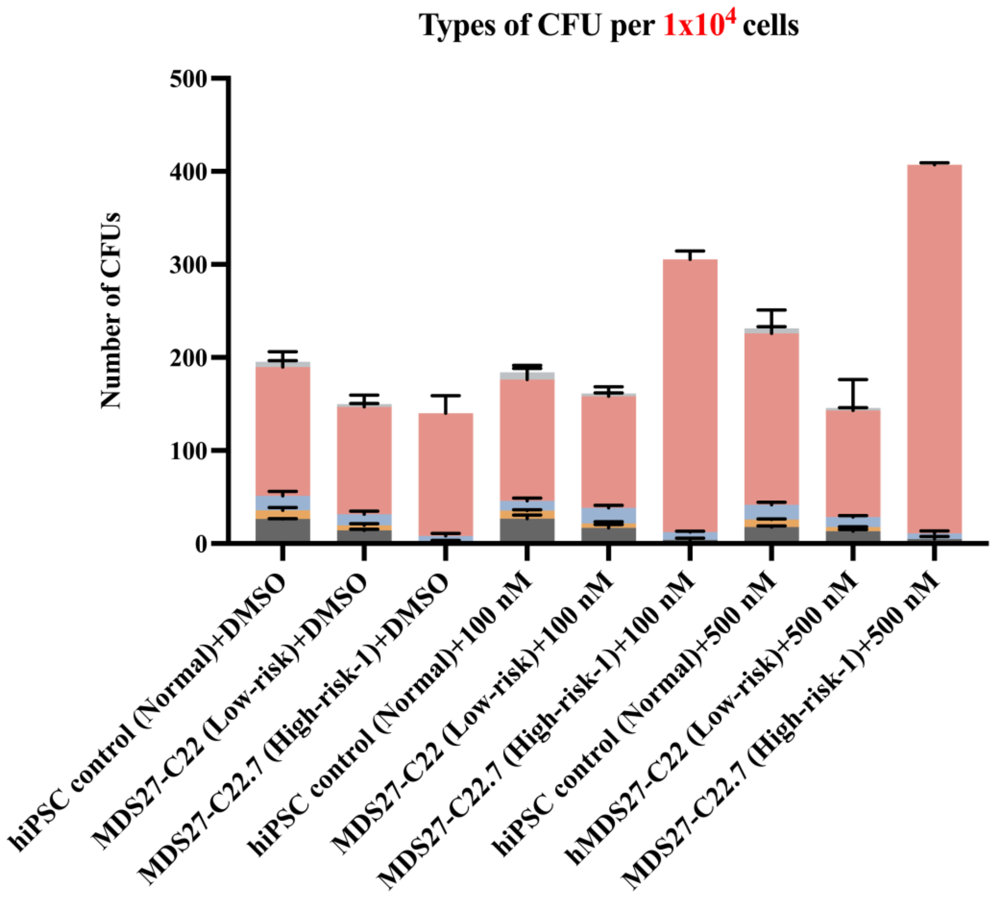

C

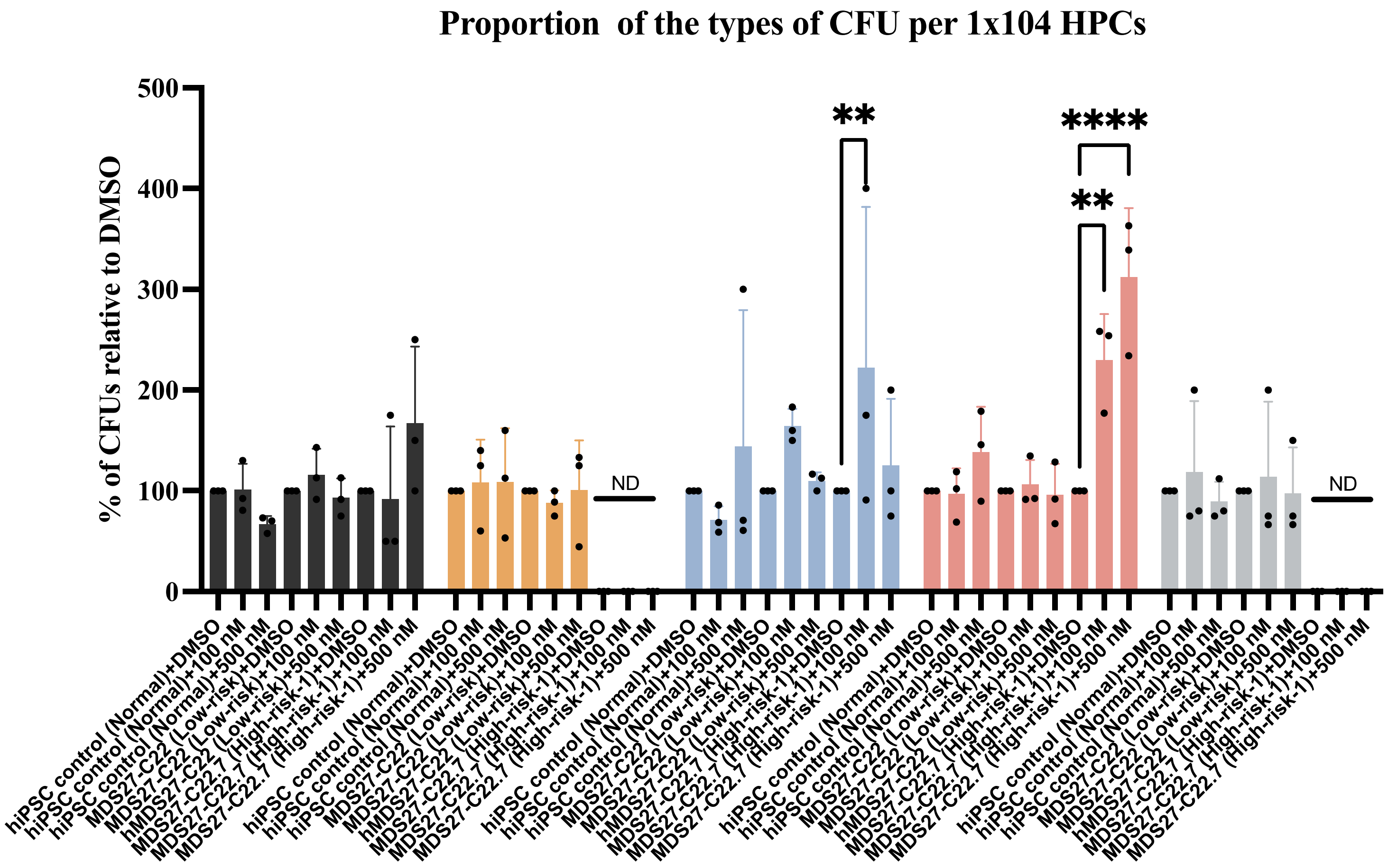

D

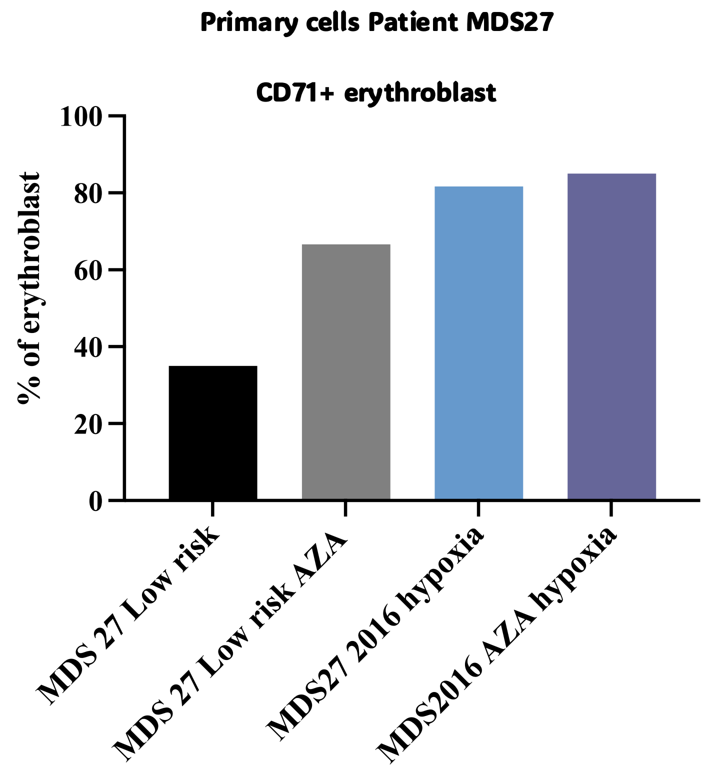

E

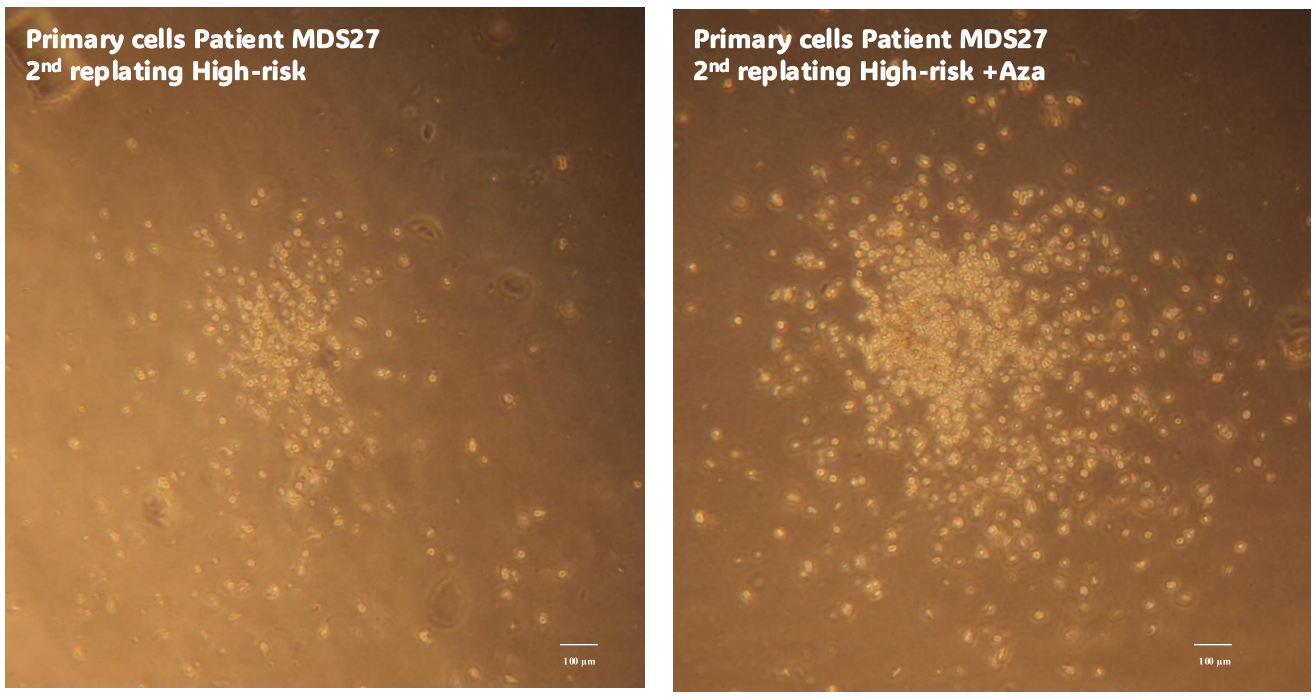

Supplementary Figure 7. High-risk MDS-iPSC containing C/EBPα mutation are sensitive to AraC and resistant to 5-Azacitine treatment

(A) Relative viability of HPC cells measured 48 hours after treatment with 200nM Ara-C. Please see Source of data file and Supplementary Data Table 5 for exact p values.

(B) Number of each type of CFUs after 14 days in semisolid medium. Mean and SEM of different lines are shown. N= 3 independent experiments.

(C) Relative percentage of each type of CFUs for 1x104 of HPCs after 14 days in semisolid media. Statistical results are presented as mean ± SEM and **** p < 0.0001, **p< 0.01, * p< 0.05 and (ns, no significant), Two-way ANOVA with Dunnet's multiple comparisons. N= 3 independent experiments. Please see Source of data file and Supplementary Data Table 5 for exact p values.

(D) Percentage of early erythroblasts (CD71+/CD235a-) in colony assays from CD34+ sorted cells from MDS27 patient samples in normoxia (before) and hypoxia (after) disease progression.

(E) Representative images illustrating the morphology of CFUs scored after 14 days in semi-solid medium (second replating). Pictures taken with primo vert microscope (Zeiss) with Canon camera at 10x magnification.

Supplementary Figure 8

A

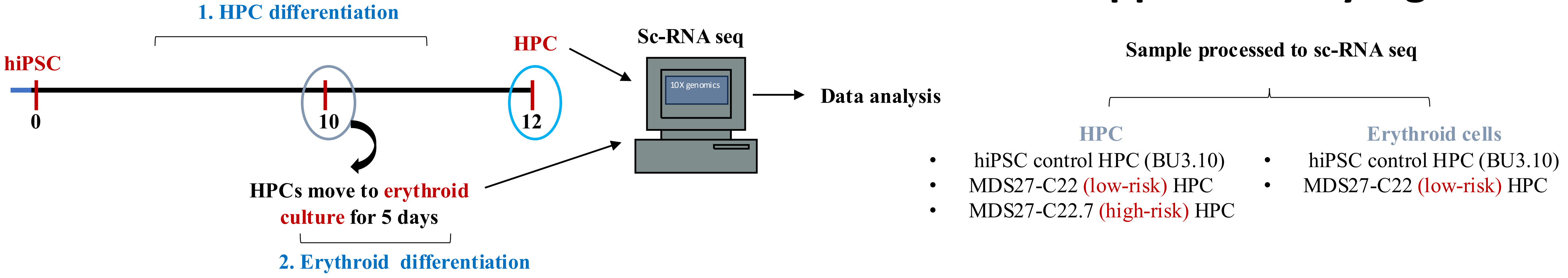

B

HPC

CC.Phase	BU3_10	MDS27_C22	MDS27_C22.7
G1	0.327088797	0.463905006	0.726658721
G2M	0.349381791	0.2131966	0.146205801
S	0.323529412	0.322898394	0.127135479

Erythoid

CC.Phase	BU3_10	MDS27_C22
G1	0.722107244	0.817739521
G2M	0.129444967	0.081586826
S	0.148447789	0.100673653

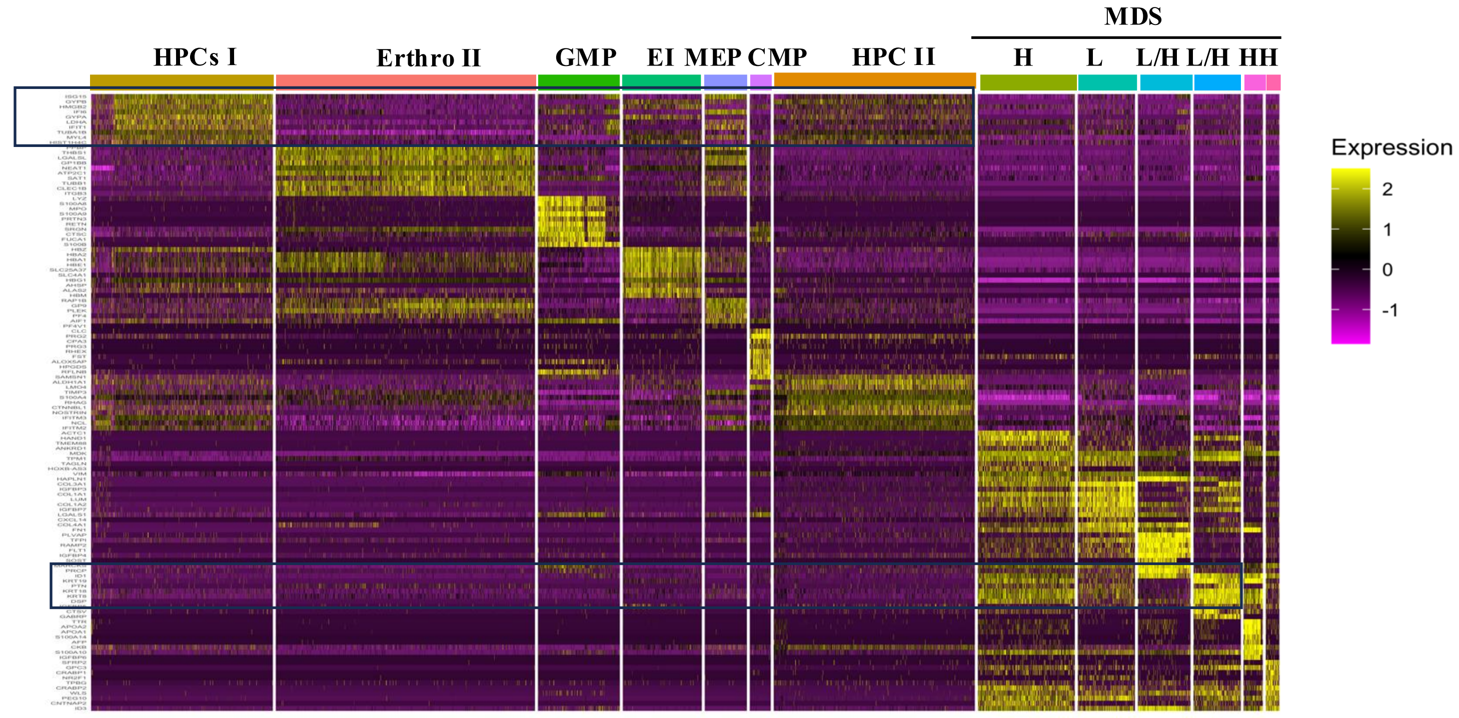

D

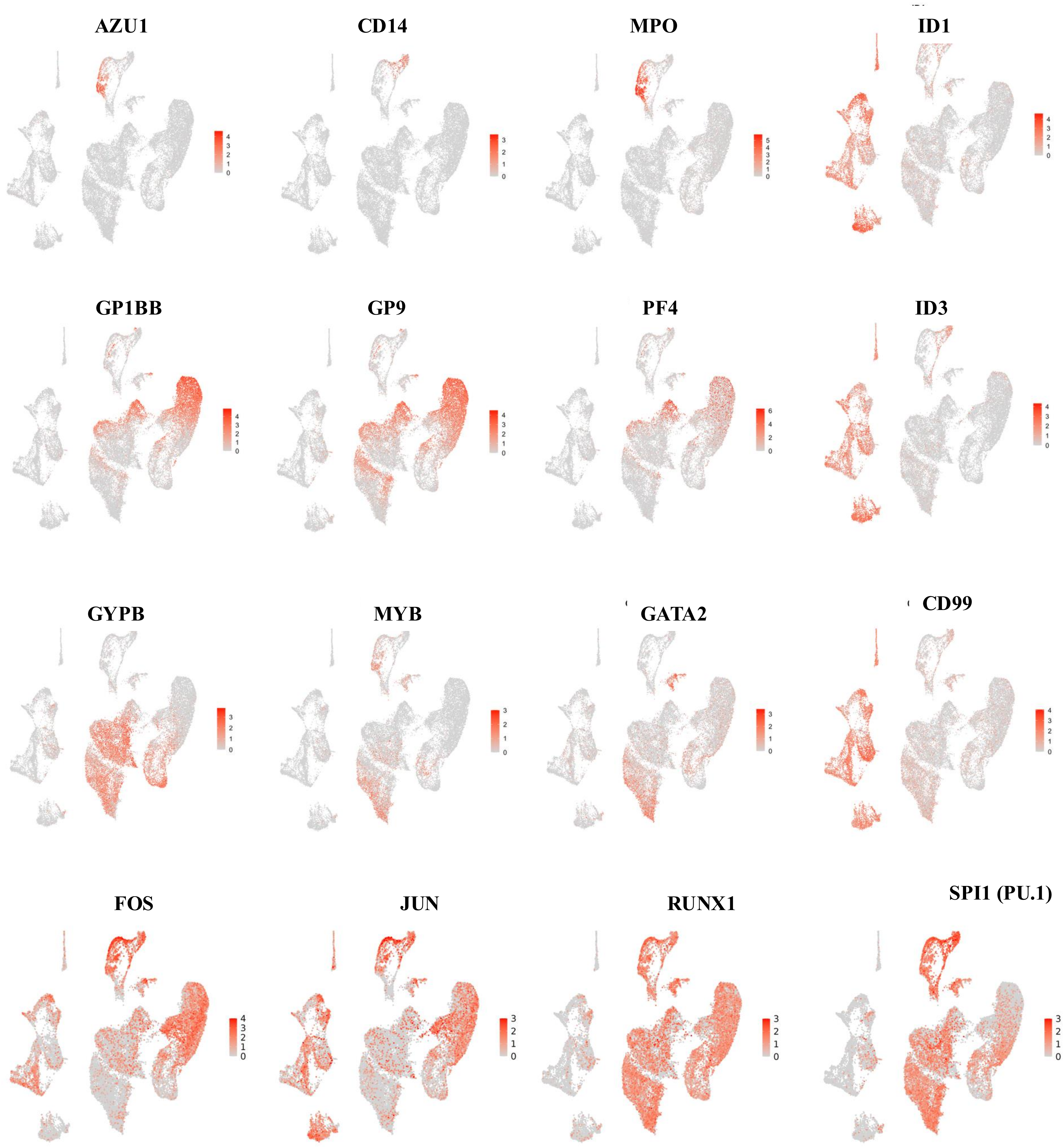

G

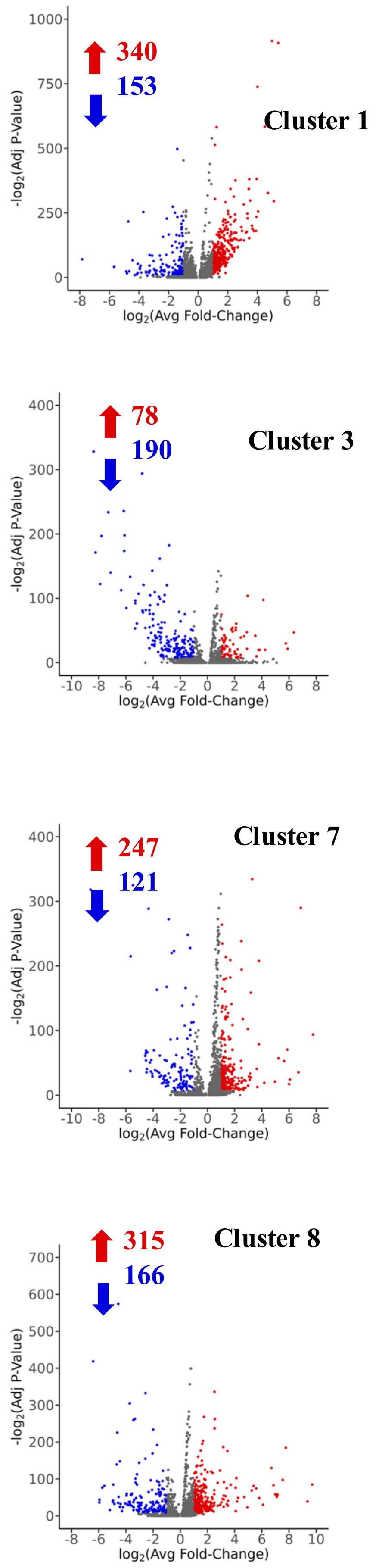

Supplementary Figure 8

E

KEGG pathways enriched in MDS low risk (MDS27-C22) compared to high risk

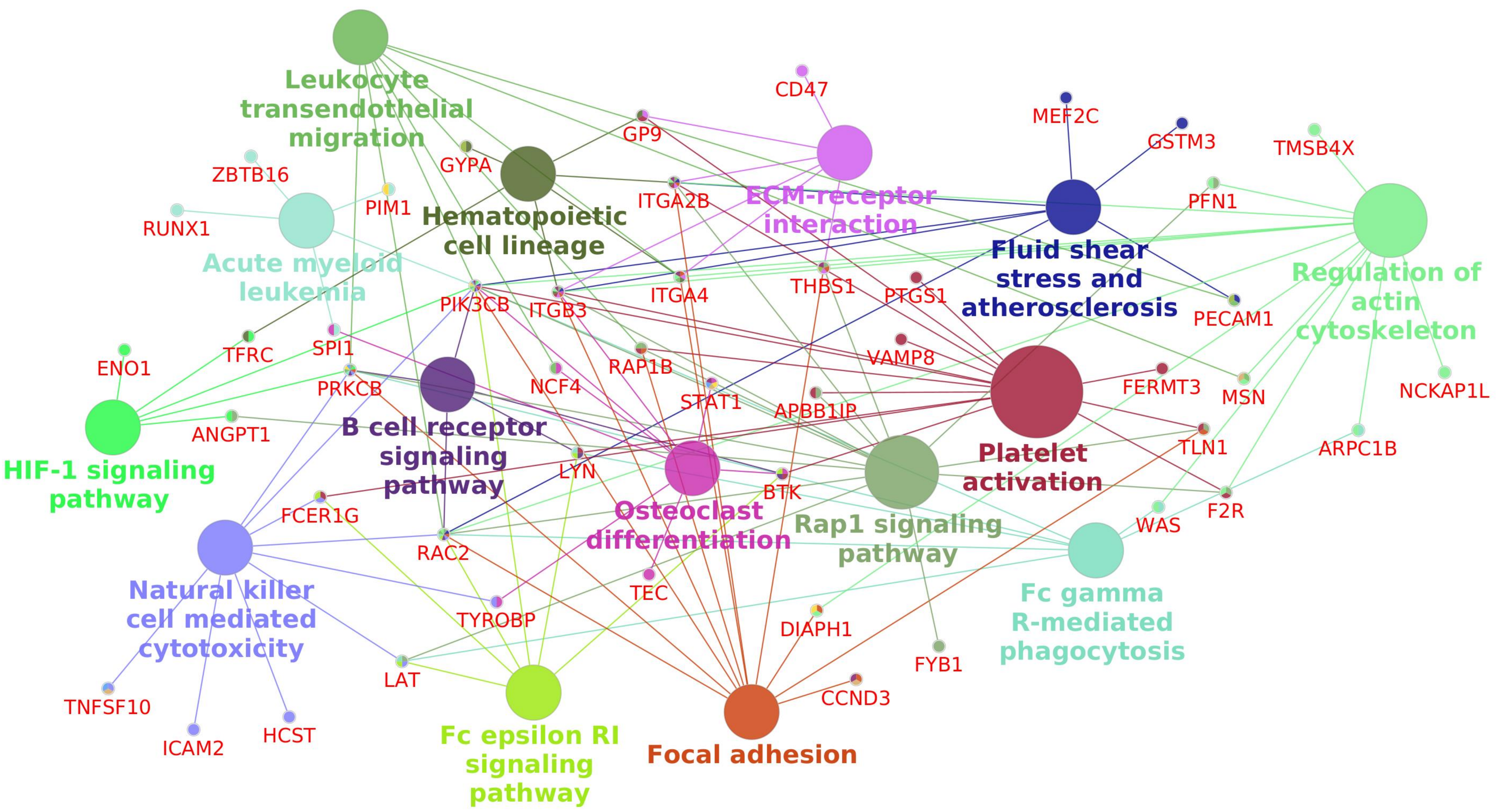

F

KEGG pathways enriched in MDS high risk (MDS27-C22.7) compared to low risk

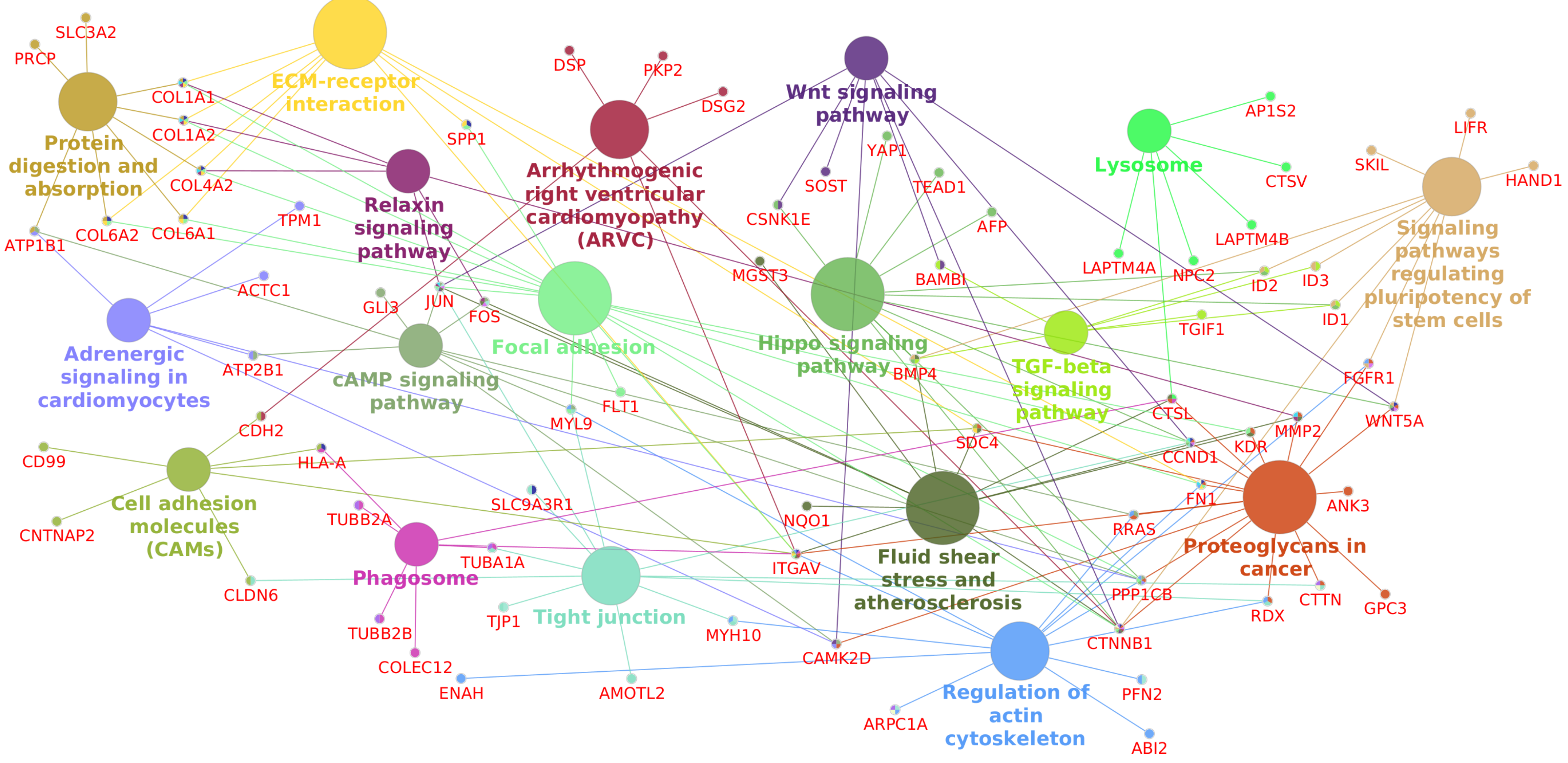

Supplementary Figure 8. Distinct MDS transcriptome signature and changes in cellular composition during disease progression measured by single-cell transcriptome analysis

(A) Schematic representation of scRNAseq procedure. Artwork generated with powerpoint Bundle-Biology.

(B) Proportion of cells in each cell cycle phase as identified by cell-cycle regulated genes.

(C) Heatmap depicting the top 10 most upregulated marker genes identified in each scRNA-seq cluster.

(D) Expression of indicated genes projected on the UMAP map. Colour intensity represents expression data log2 normalized unique molecular identifier (UMI) counts.

(E) KEGG pathway enrichment analysis of genes upregulated in Low risk MDS27-C22 iPSC compared to high risk MDS27-C22.7 iPSC from Pseudo-bulk RNA-seq.

(F) KEGG pathway enrichment analysis of genes upregulated in high risk MDS27-C22.7 compared to Low risk MDS27-C22 iPSC from Pseudo-bulk RNA-seq.

(G) Volcano plots for the indicated clusters showing the number of DGE of high risk MDS27-C22.7 compared to Low risk MDS27-C22. A gene was considered to be differentially expressed if it had a fold-change greater than 2 (log2 fold-change >1) and an adjusted p-value < 0.01.

Supplementary Table 1. Episomal reprogramming media

Medium	Component	Final concentration	Company	Cat. number
PBMNCs expansion medium	a-mem	Base	Merch	M8042
	HIFBS	10%	Sigma	12106C
	IL-3	10 ng/ml	Peprotech	200-03
	IL-6	10 ng/ml	Peprotech	200-06
	G-CSF	10 ng/ml	Peprotech	300-23
	GM-CSF	10 ng/ml	Peprotech	300-03
Human ESC medium	DMEM/F12	Base	Gibco	11320033
	Knock out Serum	20%	Gibco	10828028
	L-Glutamine	1 mM	Gibco	25030024
	Non-essential amino acids	1 mM	Gibco	11140035
	2-mercaptoethanol	0.1M	Sigma	M7522
	bFGF	10 ng/ml	Peprotech	100-18
