# Supplementary material for: A heterozygous CEBPA mutation disrupting the bZIP domain in a RUNX1 and SRSF2 mutational background causes MDS disease progression
Source: Nat Commun. 2025 Jul 1;16:5489. doi: 10.1038/s41467-025-60192-8 (PMC12219322; doi:10.1038/s41467-025-60192-8)
Supplement: Supplementary file 2 — Description of Additional Supplementary Files [file 41467_2025_60192_MOESM2_ESM.pdf]

Description of Additional Supplementary Files:

Supplementary Data 1: Genotyping of clones generated by Sendai virus and episomal reprograming. Genotyping was performed by the Hospital La Fe, Valencia, Spain for generated clones from MDS27 peripheral blood sample on 2013, using an array of the 40 most mutated genes in AML. All clones harbour the same mutations as the original sample (2013).

Supplementary Data 2: Pseudo-bulk cluster analysis. Differential gene expression analysis comparing all cells from MDS27-C22.7 (High Risk) to MDS27-C22 (Low Risk) samples. P-Values were calculated using a two-sided Wilcoxon Rank Sum test using the FindMarkers function in Seurat. The resulting pvalues were corrected for multiple testing using the Bonferroni method.

Supplementary Data 3: DEG and GO terms for MDS clusters. Differential gene expression analysis comparing cells from MDS27-C22.7 (High Risk) to MDS27-C22 (Low Risk) samples within clusters 1, 3, 7 and 8. P-Values were calculated using a two-sided Wilcoxon Rank Sum test using the FindMarkers function in Seurat. The resulting p-values were corrected for multiple testing using the Bonferroni method. Enriched GO terms were found using the list of significantly differentially expressed genes (log2 foldchange > 1; adjusted p-value < 0.01) using DAVID. P-values were calculated using a 1-sided Fisher's exact test as applied in DAVID and corrected for multiple testing using the Benjamini-Hochberg method.

Supplementary Data 4: Myeloid signature for sc-RNAseq myeloid clusters. Differential gene expression analysis comparing MDS27-C22 to BU3.10 cells. P-Values were calculated using a two-sided Wilcoxon Rank Sum test using the FindMarkers function in Seurat. The resulting p-values were corrected for multiple testing using the Bonferroni method.

Supplementary Data 5: Statistical analysis and exact p values for all Figures. Artwork generated with powerpoint Bundle-Biology: scienceppt.com/b/science-ppt-bundle-biology.
