# Supplementary material for: A heterozygous CEBPA mutation disrupting the bZIP domain in a RUNX1 and SRSF2 mutational background causes MDS disease progression
Source: Nat Commun. 2025 Jul 1;16:5489. doi: 10.1038/s41467-025-60192-8 (PMC12219322; doi:10.1038/s41467-025-60192-8)
Supplement: Supplementary file 8 — Reporting Summary [file 41467_2025_60192_MOESM8_ESM.pdf]

nature portfolio

Corresponding author(s): Paloma Garcia
Last updated by author(s): 05/05/2025

Reporting Summary

Nature Portfolio wishes to improve the reproducibility of the work that we publish. This form provides structure for consistency and transparency in reporting. For further information on Nature Portfolio policies, see our Editorial Policies and the Editorial Policy Checklist.

Statistics

For all statistical analyses, confirm that the following items are present in the figure legend, table legend, main text, or Methods section.

n/a	Confirmed	
☐	☒	The exact sample size (*n*) for each experimental group/condition, given as a discrete number and unit of measurement
☐	☒	A statement on whether measurements were taken from distinct samples or whether the same sample was measured repeatedly
☐	☒	The statistical test(s) used AND whether they are one- or two-sided<br>*Only common tests should be described solely by name; describe more complex techniques in the Methods section.*
☒	☐	A description of all covariates tested
☐	☒	A description of any assumptions or corrections, such as tests of normality and adjustment for multiple comparisons
☐	☒	A full description of the statistical parameters including central tendency (e.g. means) or other basic estimates (e.g. regression coefficient) AND variation (e.g. standard deviation) or associated estimates of uncertainty (e.g. confidence intervals)
☒	☐	For null hypothesis testing, the test statistic (e.g. *F*, *t*, *r*) with confidence intervals, effect sizes, degrees of freedom and *P* value noted<br>*Give P values as exact values whenever suitable.*
☒	☐	For Bayesian analysis, information on the choice of priors and Markov chain Monte Carlo settings
☒	☐	For hierarchical and complex designs, identification of the appropriate level for tests and full reporting of outcomes
☒	☐	Estimates of effect sizes (e.g. Cohen's *d*, Pearson's *r*), indicating how they were calculated

Our web collection on statistics for biologists contains articles on many of the points above.

Software and code

Policy information about availability of computer code

Data collection	Images for pluripotent markers, chromosmes and DiffKuick staining were acquired with Leica DM6000 light microscope and for Alkaline phosphatase and cells in culture using a primo vert microscope (ZEISS).<br>Flow cytometry data was obtained using a FORTESSA and sorting using a BD FACSAriaTM Fusion (BD bioscience).
Data analysis	Statiscal data analysis was perfromed using PRISM. The statistical analysis and exact p value for each figure has been compiled in Supplementary Data Table 5.<br><br>ATAC-seq: Raw sequencing reads were processed to remove low-quality bases and Nextera ATAC adapter sequences using Trimmomatic v0.39 93. Reads were then aligned to the human genome (version hg38) using Bowtie2 v2.2.594 with the --very-sensitive-local parameter. Potential PCR duplicates were removed from the alignments using Picard MarkDuplicates v2.16.10 (http://broadinstitute.github.io/picard). Peaks were called using MACS2 v2.2.7.195 with the options --nomodel --call-summits -q 0.05 -B --trackline. The resulting peaks were then filtered to remove peaks found in the hg38 blacklist96 and to retain only peaks that had a summit height greater than 5 in both replicates of either low- or high-risk MDS clones. Peaks were then combined to produce a single peak union using the merge command in BedTools v2.30.0 97. Read counts were obtained using featureCounts v2.0.1 98 and normalized using edgeR v3.36.0 99 in R v4.1.2. Differentially accessible peaks were identified using the voom method in the Limma package v3.50.3 100. A peak was considered to be differentially accessible if it had a fold-difference of at least 1.5 between conditions. Motif enrichment analysis was carried out in the sets of differentially accessible peaks using the findMotifsGenome.pl function in Homer v4.9.1 101 with the options -size 200 -noknown. To create read density plots, peaks were first ranked according to their fold-difference between low- and high-risk MDS cells. Read densities were then calculated in a 2kb window centered on the peak summit using the annotatePeaks.pl function in Homer with the options -size 2000 -hist 10 -ghist -bedGraph and the bedGraph files produced by MACS2. These were then plotted as a heatmap using Java TreeView v1.1.6r4 102.

scRNAseq:Reads from single-cell RNA-Seq experiments were aligned to the human genome (version hg38) and quantified using the count function in CellRanger v4.0.0 from 10x Genomics and using gene models from Ensembl as the reference transcriptome. Single-cell analysis was carried out using the Seurat package v4.1.0103 in R v4.1.2. The data was first filtered to remove cells which had less than 500 or more than 5000 expressed genes detected, as well as those which had more than 20% of reads aligned to mitochondrial transcripts. The count data from the individual samples were then combined into a single dataset, which was then normalized and scaled using the NormaliseData and ScaleData functions in Seurat, using the top 2000 most variable genes. Clustering was then performed by first conducting a Principal Components Analysis (PCA) and selecting the top 26 principal components for further analysis. The clusters were then identified using the FindClusters function in Seurat using a resolution value of 0.3.
Single-cell trajectory analysis was carried out using Monocle3 v0.2.3 104. Processed data from Seurat was imported into Monocle using the as.cell_data_set command in SeuratWrappers (https://github.com/satijalab/seurat-wrappers). Trajectories were then inferred using the learn_graph command in Monocle. Pseudotime was calculated using the order_cells command, using the earliest inferred HSC population as the root node. These were then plotted using the UMAP coordinates calculated by Seurat.

For manuscripts utilizing custom algorithms or software that are central to the research but not yet described in published literature, software must be made available to editors and reviewers. We strongly encourage code deposition in a community repository (e.g. GitHub). See the Nature Portfolio guidelines for submitting code & software for further information.

Data

Policy information about availability of data

All manuscripts must include a data availability statement. This statement should provide the following information, where applicable:

- Accession codes, unique identifiers, or web links for publicly available datasets
- A description of any restrictions on data availability
- For clinical datasets or third party data, please ensure that the statement adheres to our policy

All data supporting the findings of this study are available within the paper and its Supplementary Information. Source data are provided with this paper. ATAC-Seq and single cell RNAseq data are available through the GEO SuperSeries accession number GEO:GSE236710.
We have also used available human gene data sets:
EPPERT_CE_HSC_LSC (gsea-msigdb.org)
EPPERT_HSC_R (gsea-msigdb.org)
EPPERT_LSC_R (gsea-msigdb.org)

JAATINEN_HEMATOPOIETIC_STEM_CELL_DN (gsea-msigdb.org)
JAATINEN_HEMATOPOIETIC_STEM_CELL_UP (gsea-msigdb.org)

GENTLES_LEUKEMIC_STEM_CELL_DN (gsea-msigdb.org)
GENTLES_LEUKEMIC_STEM_CELL_UP (gsea-msigdb.org)

VALK_AML_WITH_CEBPA (gsea-msigdb.org)
VALK_AML_CLUSTER_4 (gsea-msigdb.org)
VALK_AML_CLUSTER_15 (gsea-msigdb.org)

Research involving human participants, their data, or biological material

Policy information about studies with human participants or human data. See also policy information about sex, gender (identity/presentation), and sexual orientation and race, ethnicity and racism.

Reporting on sex and gender	male
Reporting on race, ethnicity, or other socially relevant groupings	NA
Population characteristics	NA
Recruitment	NA

Ethics oversight	Human patients with MDS were recruited from the clinic held at the Centre for Clinical Haematology, University Hospital Birmingham NHS Foundation Trust. Patient MDS27 have read the patient information sheet and signed the consent form. The study was conducted as according to Good Clinical Practice guidelines, consistent with the principles that have their origin in the Declaration of Helsinki. The study was approved by the West Midlands – Solihull Research Ethics Committee (10/H1206//58).

Note that full information on the approval of the study protocol must also be provided in the manuscript.

Field-specific reporting

Please select the one below that is the best fit for your research. If you are not sure, read the appropriate sections before making your selection.

[x] Life sciences   [ ] Behavioural & social sciences   [ ] Ecological, evolutionary & environmental sciences

For a reference copy of the document with all sections, see nature.com/documents/nr-reporting-summary-flat.pdf

Life sciences study design

All studies must disclose on these points even when the disclosure is negative.

Sample size	In vitro: No sample size calculation was performed due to restricted number of available samples. A sample size of 3 to 4 biological replicates per discrete experiment was performed.<br>In vivo: No sample size calculation was performed due to the restricted number of available samples. Pilot experiment as shown in the manuscript indicated successful engraftment.
Data exclusions	No data was excluded except for scRNAseq.<br>Single-cell RNA-seq data were excluded based on quality control criteria, as described in the Methods.
Replication	In vitro: Data is representative of 3 to 4 biological replicates. Experiments displayed in manuscript are single independent. Experiments were performed in at least 2 clones of the same hiPSC<br>In vivo: Three different transplantation routes (Intravenous,IV; Intra-bone,IB; Scaffolds) were tested. For IV and IB experiments 11 animals were transplanted. For humanised scaffolds experiments, 17 scaffolds were implanted in 6 mice.
Randomization	Cell samples and animals were allocated randomly. Animals of the same litter were distributed equally among all groups.
Blinding	Blinding was not applicable or logistically possible in our study.

Reporting for specific materials, systems and methods

We require information from authors about some types of materials, experimental systems and methods used in many studies. Here, indicate whether each material, system or method listed is relevant to your study. If you are not sure if a list item applies to your research, read the appropriate section before selecting a response.

Materials & experimental systems

n/a	Involved in the study	
[ ]	[x]	Antibodies
[ ]	[x]	Eukaryotic cell lines
[x]	[ ]	Palaeontology and archaeology
[ ]	[x]	Animals and other organisms
[ ]	[x]	Clinical data
[x]	[ ]	Dual use research of concern
[x]	[ ]	Plants

Methods

n/a	Involved in the study	
[x]	[ ]	ChIP-seq
[ ]	[x]	Flow cytometry
[x]	[ ]	MRI-based neuroimaging

Antibodies

Antibodies used	Immunofluorescence:<br>SOX2 (AF2018, clone 245610, R&D); NANOG (AF1997, clone ABZ92376, R&D). secondary Ab goat anti-mouse-IgM-Alexa, Fluor 488 (A10684, Invitrogen) and goat anti-mouse secondary antibody (A21052, Invitrogen). TRA1-81 mouse primary Ab (MA1-024, Clone C1.261, Invitrogen) .<br><br>Flow cytometry:<br>Fc receptor binding inhibitor (Fc block) (14916173, Clone AB468581, eBioscience).<br>CD43-APC (560198, clone 1G10, BDPharmingen),

CD45-FITC (11045942, clone HI30, eBioscience)
CD34-PE (550619, clone 8G12, BD Pharmingen).
Isotype controls mouse IgG1k-PE (clone MOPC-31C, BD Pharmingen) and mouse IgG1k-FITC/APC (clone P.3.6.2.8.1, Thermofisher).
CD71-APC (17071941, clone OKT-9, eBioscience) glycophorin A (CD235a-PE) (12-9987-80, clone GA-R2, eBioscience).
Isotype controls mouse IgG1k- APC (clone P.3.6.2.8.1, Thermofisher) and mouse IgG2bk-PE (clone Ebmg2b, Thermofisher)
CD14-APCCy7 (47014942, clone 61D3, eBioscience) and CD11b- PECy7 (15518356, clone ICRF44, eBioscience).
Isotype controls mouse IgG1k- APCCy7/PECy7 (clone P.3.6.2.8.1, Thermofisher)

Xenotransplants:
human or murine antigens [mCD45-PerCPCy5.5 (Clone 30-F11, eBioscience), hCD45-APCef780 (Clone HI30, eBioscience), hCD33-APC (Clone WM53, BD Pharmingen), hCD3-PE (Clone UCHT1, BD Pharmingen), and hCD19-PE (Clone HIB19, BD Pharmingen).

Validation

Fc receptor binding inhibitor (14916173, Clone AB468581, eBioscience). Tested by manufacturer. Purified human Fc gammaR-binding inhibitor has been pre-titrated and tested by inhibiting binding of fluorochrome-conjugated isotype controls to U937 cells. This can be used at 20 µL per test. A test is defined as the amount (µg) of antibody that will stain a cell sample in a final volume of 100 µL.
CD43-APC (560198, clone 1G10, BDPharmingen): Borche L, Lozano F, Vilella R, Vives J. CD43 monoclonal antibodies recognize the large sialoglycoprotein of human leukocytes. Eur J Immunol. 1987; 17(10):1523-1526. (Biology) Kishimoto T, von dem Borne AEG, Goyert SM,et al., ed. Leucocyte Typing VI: White Cell Differentiation Antigens. London: Garland Publishing; 1997. (Clone-specific) Knapp W, Dorken B, Rieber EP, et al, ed. Leucocyte Typing IV. New York: Oxford University Press; 1989:1-1208. (Biology) Schlossman SF, Boumsell L, Gilks W, et al, ed. Leukocyte Typing V: White Cell Differentiation Antigens. New York: Oxford University Press; 1995. (Biology
CD45-FITC (11045942, clone HI30, eBioscience). Tested by manufacturer: This Antibody was verified by Cell treatment to ensure that the antibody binds to the antigen stated.
CD34-PE (550619, clone 8G12, BD Pharmingen). 52 citations in manufacturers webpage.
CD71-APC (17071941, clone OKT-9, eBioscience): This Antibody was verified by Knockout to ensure that the antibody binds to the antigen stated.
glycophorin A (CD235a-PE) (12-9987-80, clone GA-R2, eBioscience). Tested by manufacturer. 42 references in manufactures webpage CD235a (Glycophorin A) Monoclonal Antibody (HIR2 (GA-R2)), PE (12-9987-82)
CD14-APCCy7 (47014942, clone 61D3, eBioscience) . 91 references, 50 published figures in manufacturers website.
CD11b- PECy7 (15518356, clone ICRF44, eBioscience). 36 references, 30 published figures in manufacturer's website.
mCD45-PerCPCy5.5 (Clone 30-F11, eBioscience), Tested by manufacturer: This Antibody was verified by Cell treatment to ensure that the antibody binds to the antigen stated.
hCD45-APCef780 (Clone HI30, eBioscience), Tested by manufacturer : This Antibody was verified by Knockout to ensure that the antibody binds to the antigen stated.
hCD33-APC (Clone WM53, BD Pharmingen). 47 citations, including Cancer Discovery on 2 June 2022 by Gambacorta; Nature Biotechnology on 1 October 2022 by Oostindie and Leukemia on 1 October 2022 by Koschade, S. E.
hCD3-PE (Clone UCHT1, BD Pharmingen), Tested by manufacturer: This Antibody was verified by Relative expression to ensure that the antibody binds to the antigen stated.
hCD19-PE (Clone HIB19, BD Pharmingen). Tested by manufacturer: This Antibody was verified by Knockout to ensure that the antibody binds to the antigen stated.

Eukaryotic cell lines

Policy information about cell lines and Sex and Gender in Research

Cell line source(s)

BU3.10 cell line: CREM003i-BU3C2; WiCell (male)
MDS27 iPSC lines generated in this work (male)

Authentication

Mutational screenong was performed to determine that the lines generated harbour the sam emutation as the patient from which they derived.

Mycoplasma contamination

Negative

Commonly misidentified lines
(See ICLAC register)

No commonly misidentified lines were used.

Animals and other research organisms

Policy information about studies involving animals; ARRIVE guidelines recommended for reporting animal research, and Sex and Gender in Research

Laboratory animals

NOD/SCID/IL2rγ−/−/IL-3/GM/SF (NSG-SGM3). Both male and female mice, aged 8 to 12 weeks, were used in this study. The Crick prioritizes the welfare of the animals used in research, providing a comfortable and stimulating environment. Crick provides various enrichment materials for the animals to keep their environment comfortable and interesting, such as nesting material, chew blocks, and interactive toys

The Crick Institute's animal facility uses a 12-hour light period followed by a 12-hour dark period to ensure a predictable and stable

environment for their animals.

The Crick Biological research facility (BRF) mice, like most laboratory mice, are typically maintained in a controlled environment with an ambient temperature range of 64-79°F (17.8-26.1°C) and a relative humidity range of 30-70%. These parameters are closely monitored and controlled within the rodent colony rooms

Wild animals	NA
Reporting on sex	Both male and female mice were used in this study.
Field-collected samples	Samples were not collected from the field.
Ethics oversight	All animal experiments were performed at the Francis Crick Institute in accordance with UK Home Office and institutional guidelines under a valid Home Office project license. Ethical approval: UK Home Office<br>License number: PLL 70/8904

Note that full information on the approval of the study protocol must also be provided in the manuscript.

Clinical data

Policy information about clinical studies
All manuscripts should comply with the ICMJEguidelines for publication of clinical research and a completedCONSORT checklist must be included with all submissions.

Clinical trial registration	NA
Study protocol	NA
Data collection	NA
Outcomes	NA

Plants

Seed stocks	NA
Novel plant genotypes	NA
Authentication	NA

Flow Cytometry

Plots

Confirm that:

- [x] The axis labels state the marker and fluorochrome used (e.g. CD4-FITC).
- [x] The axis scales are clearly visible. Include numbers along axes only for bottom left plot of group (a 'group' is an analysis of identical markers).
- [x] All plots are contour plots with outliers or pseudocolor plots.
- [x] A numerical value for number of cells or percentage (with statistics) is provided.

Methodology

Sample preparation	The cells were pelleted by centrifugation at 300g for 5 minutes, the supernatant was carefully removed, and the cells were resuspended in 100µL of sPBS/2%FBS. Cells were incubated with Fc block antibody for 45-60 minutes on ice. Surface marker staining cocktail or isotype control was then added. The unstained sample was resuspended in 100µL of 1X DPBS (Thermo Scientific, 14190250) and viability dye (Thermo Scientific, 65-0865-14) control was resuspended in 100µL of 1X DPBS containing the viability dye. (1:2000) The samples were incubated on ice for 1 hour, washed with

	1mL of PBS /2%FBS and pelleted by centrifugation at 300g for 5 minutes. Samples were resuspended in 300 µL of PBS/2%FBS and then analyzed on the Fortessa. FCS files were exported and analyzed using FlowJo software.
Instrument	Fortessa (BD Biosciences, Oxford, UK).
Software	Analysis using Flowjo.
Cell population abundance	25,000-50,000 cells were sorted cells for ATAC-seq studies with over 95% purrity.<br>For CRISPR Cas9 sorted GFP+ populations varied between 10,000 to 40,000. No purity checked was performed as indicvidual clones needed to be anlaysed.
Gating strategy	Preliminary gate: FSC-A x SSC-A -> Single Cells: FSC-W x FSC-H -> Viability Gate: Viability Dye x SSC-A -> CD71/CD235a or CD11b/CD14 or CD43/CD34 or CD45/CD34 or CD33/CD11b gatings.<br>All gates set using Unstained Control

☒ Tick this box to confirm that a figure exemplifying the gating strategy is provided in the Supplementary Information.
